# A lipid-binding loop of botulinum neurotoxin serotypes B, DC and G is an essential feature to confer their exquisite potency

**DOI:** 10.1371/journal.ppat.1007048

**Published:** 2018-05-02

**Authors:** Daniel Stern, Jasmin Weisemann, Alexander Le Blanc, Laura von Berg, Stefan Mahrhold, Janett Piesker, Michael Laue, Peter B. Luppa, Martin Bernhard Dorner, Brigitte Gertrud Dorner, Andreas Rummel

**Affiliations:** 1 Biological Toxins (ZBS 3), Centre for Biological Threats and Special Pathogens, Robert Koch Institute, Berlin, Germany; 2 Institut für Toxikologie, Medizinische Hochschule Hannover, Hannover, Germany; 3 Institute for Clinical Chemistry and Pathobiochemistry, Klinikum rechts der Isar, Technische Universität München, München, Germany; 4 Advanced Light and Electron Microscopy (ZBS 4), Centre for Biological Threats and Special Pathogens, Robert Koch Institute, Berlin, Germany; University of Pittsburgh School of Medicine, UNITED STATES

## Abstract

The exceptional toxicity of botulinum neurotoxins (BoNTs) is mediated by high avidity binding to complex polysialogangliosides and intraluminal segments of synaptic vesicle proteins embedded in the presynaptic membrane. One peculiarity is an exposed hydrophobic loop in the toxin’s cell binding domain H_C_, which is located between the ganglioside- and protein receptor-binding sites, and that is particularly pronounced in the serotypes BoNT/B, DC, and G sharing synaptotagmin as protein receptor. Here, we provide evidence that this H_C_ loop is a critical component of their tripartite receptor recognition complex. Binding to nanodisc-embedded receptors and toxicity were virtually abolished in BoNT mutants lacking residues at the tip of the H_C_ loop. Surface plasmon resonance experiments revealed that only insertion of the H_C_ loop into the lipid-bilayer compensates for the entropic penalty inflicted by the dual-receptor binding. Our results represent a new paradigm of how BoNT/B, DC, and G employ ternary interactions with a protein, ganglioside, and lipids to mediate their extraordinary neurotoxicity.

## Author summary

Botulinum neurotoxins are Janus-faced molecules: due to their exquisite toxicity, botulinum neurotoxins are considered as biological weapons, but they are also highly effective medicines for numerous neurological indications. However, what mediates their exquisite toxicity? The exclusive binding to neurons and the subsequent paralysis cuts off the host’s communication networks. The neurospecific binding is ensured by anchoring to two receptor molecules both embedded in the membrane: a complex ganglioside and a synaptic vesicle protein. Here, we reveal a third interaction between a hydrophobic so-called H_C_ loop protruding from the surface of the serotypes BoNT/B, DC, and G into the lipid membrane. Only this H_C_ loop ensures their high-affinity binding to the neuronal receptors also at physiological temperature (37°C). Hereby, BoNT/B, DC, and G prevent untimely dissociation prior to uptake into the neuron. Therefore, our study provides the mechanistic basis for the development of inhibitors to combat botulism, but it also has implications for engineering toxin—membrane interactions yielding optimized BoNT-based therapeutics to treat neuromuscular dysfunctions successfully. Intriguingly, a broadly neutralizing anti-HIV-1 antibody shares a similar strategy, emphasizing the general relevance of our results for host—pathogen interactions.

## Introduction

Botulinum neurotoxins (BoNTs) are the most toxic bacterial toxins known and are produced e.g. in food by the anaerobic, spore-forming bacteria *Clostridium (C*.*) botulinum*, *C*. *butyricum*, and *C*. *baratii*. When contaminated food is ingested, BoNTs specifically inhibit acetylcholine release at the neuromuscular junctions. The resulting flaccid paralysis called botulism can lead to death by respiratory failure [1]. Due to their extraordinary toxicity (intraperitoneal median lethal dose (LD_50_): 1 ng/kg [2]), BoNTs are regarded as a potential biothreat agent [3]. On the other hand, the BoNTs are successfully exploited as pharmacological agents for a broad range of medical and cosmetic applications [4]. Both their potency and specificity can be attributed to an elaborate and elegant mode of action, mediated by the different domains of the 150 kDa molecule [5]. First, the 50 kDa C-terminal domain H_C_ of the 100 kDa heavy chain (HC) mediates high-affinity binding to specific receptors on the presynaptic membrane. Next, BoNT is taken up into recycling synaptic vesicles whereupon acidification causes the 50 kDa N-terminal domain (H_N_) to form a pore through which the 50 kDa light chain (LC) is translocated into the cytoplasm. Finally, LC specifically cleaves different members of the soluble N-ethylmaleimide-sensitive-factor attachment receptor (SNARE) protein complex which inhibits fusion of neurotransmitter-filled vesicles at cholinergic synapses. Until now, seven established BoNT serotypes (BoNT/A-G) and the newly pronounced BoNT/HA [aka BoNT/H or BoNT/FA], BoNT/X, and eBoNT/J [aka BoNT/En]) with more than 40 subtypes have been described which differ by the usage of their specific receptors, their substrate recognition, and/or specific cleavage site targeted [6–9].

**Fig 1:**
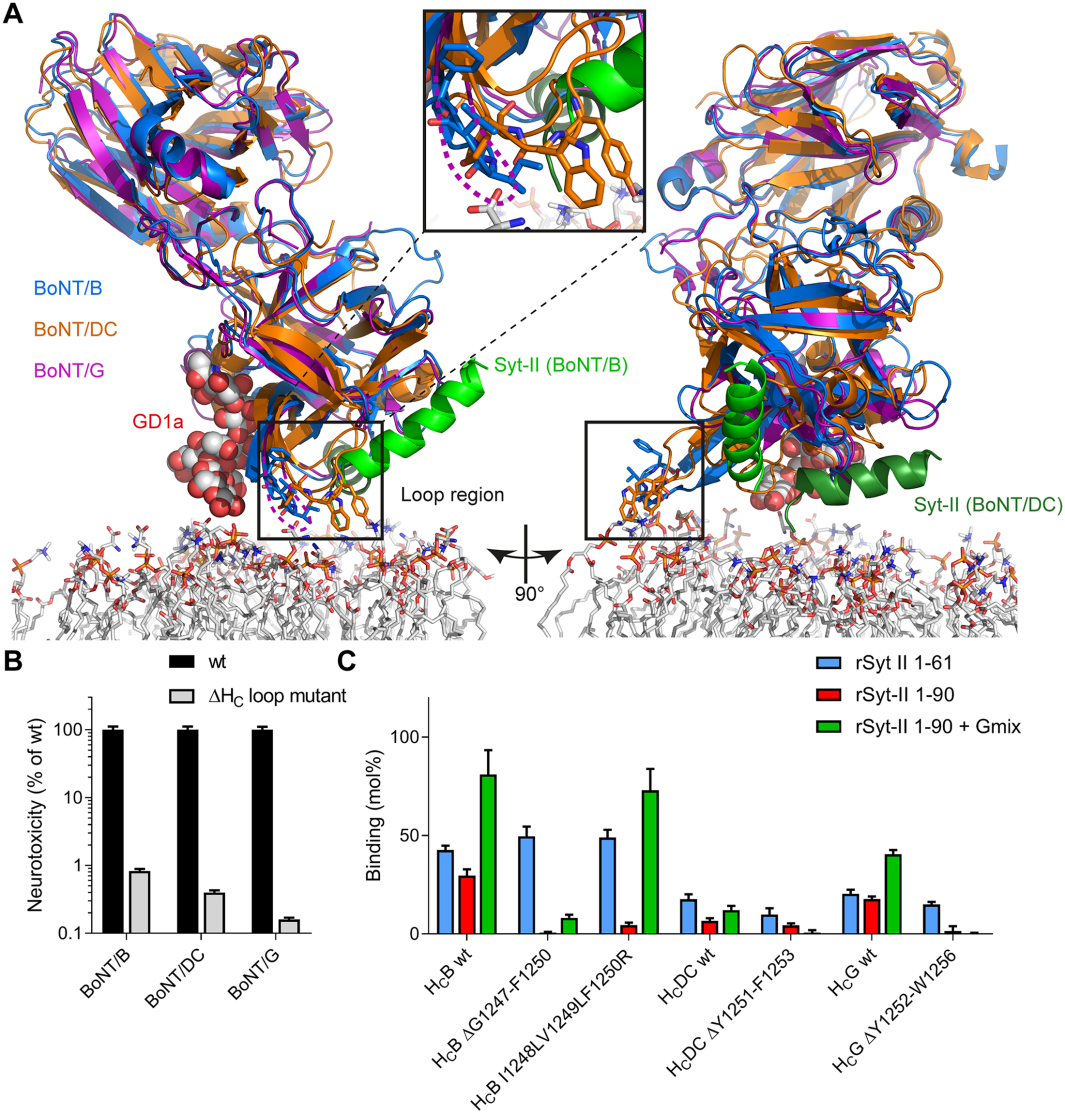
A hydrophobic loop is located between the synaptotagmin- and ganglioside-binding site of BoNT/B, DC, and G which confers high-affinity receptor binding and high toxicity of BoNT/B, DC, and G. **A** Superposition of dual-receptor binding of BoNT/B (blue ribbon; PDB IDs 1EPW [55] and 4KBB [11]) to ganglioside GD1a (space fill model) and synaptotagmin-II (green ribbon), BoNT/G (pink ribbon PDB ID 2VXR [40]), and the interaction between BoNT/DC (magenta ribbon) and synaptotagmin-II (dark green ribbon side view; PDB ID 4ISR [22]) in proximity to a model membrane. Key amino acids of the hydrophobic loop (B: G1247-F1250, DC: Y1251-F1253; black box and insert) located between the ganglioside and synaptotagmin-binding sites are shown as stick models. **B** Determination of neurotoxicity of BoNT ΔH_C_ loop vs wild-type in MPN assay. The neurotoxicity of single chain (sc) scBoNTBSL ΔG1247-F1250 (n = 4), scH6tBoNTDCS ΔY1251-F1253 (n = 4), and BoNTGS ΔY1252-W1256 (n = 2) was determined in comparison to the corresponding BoNT wild-type using dose-response curves (data are represented as mean ± SD). **C** GST pull down of wild-type and ΔH_C_ loop-mutants of H_C_B, H_C_DC, and H_C_G by GST-synaptotagmin-II with or without gangliosides in Triton X-100 micelles. Binding of 100 pmol of the wild-type or ΔH_C_ loop H_C_ fragments to 150 pmol GST-rSyt-II 1–61, 1–90, or 1–90 in 20 mM Tris pH 8, 80 mM NaCl, 0.5% Triton X-100 in the presence of 125 μg of ganglioside mix (Gmix) embedded in Triton X-100 micelles immobilized to glutathione-sepharose 4B matrix and subsequent SDS-PAGE analysis (see also S2 Fig; data are represented as mean ± SD, n = 3).

According to the current dual-receptor binding paradigm, the simultaneous interaction with a protein and a carbohydrate receptor is needed for high-affinity binding of most serotypes [10–13]. While polysialogangliosides constitute the carbohydrate receptors, the luminal domains of different isoforms of the synaptic vesicle proteins SV2 (SV2A, B, C) and synaptotagmin (Syt-I or II) were identified as the protein receptors (reviewed in [14]). Here, BoNT/A and E bind to SV2 [13, 15–21] whereas BoNT/B, G, and the mosaic serotype BoNT/DC bind to Syt-I and II [11, 12, 22–28]. For BoNT/D and F, the functional details of the contribution of SV2 to receptor binding still need to be elucidated [29–31]. No protein receptor has been identified for BoNT/C [32, 33], BoNT/X, and eBoNT/J so far. Instead, binding of BoNT/C is mediated by two independent ganglioside-binding sites (GBS) and an interjacent WY-loop rich in aromatic residues [34–37]. A similar loop called ganglioside-binding loop (GBL) is present in BoNT/DC, and a role in binding to isolated ganglioside GM1a has been demonstrated [35]. The mutations of the three aromatic residues Y1251, W1252, and F1253 to alanine in the GBL region as well as GBL deletion equally abolished binding to GM1a, GD1a, and GQ1b embedded into a 2-oleoyl-1-palmitoyl-sn-glycero-3-phosphocholine (POPC) monolayer and binding and uptake into P19-derived neurons [38]. Lately, identical H_C_DC mutants were shown to lack binding to liposome-embedded ganglioside mix, but also to phosphatidylcholine-only liposomes, while H_C_DC wild-type exhibited weak binding [39]. Interestingly, the well-characterized Syt-binders BoNT/B and G also exhibit hydrophobic loops at analogous positions (Fig 1A) which we will call ‘H_C_ loop’ in this work. Contributions of their H_C_ loops to membrane binding were hypothesized but have never been shown experimentally [23, 24, 40].

In this work, we generated recombinant full-length BoNTs and isolated receptor-binding domains H_C_ of BoNT/B, DC, and G devoid of key residues in their H_C_ loops and analyzed the contribution of the H_C_ loop to both binding and toxicity. Here, markedly reduced toxicities of BoNT ΔH_C_ loop mutants indicate a key role for the H_C_ loop in mediating the high toxicity. Systematic surface plasmon resonance (SPR) measurements revealed that binding of H_C_B, H_C_DC, and H_C_G ΔH_C_ loop mutants to isolated Syt-II remains unaltered, while stable binding to gangliosides or Syt-II incorporated into micelles and/or nanodiscs is lost. The low-affinity binding of H_C_B, H_C_DC, and H_C_G ΔH_C_ loop mutants towards dual-receptor nanodiscs containing both Syt-II protein and GT1b ganglioside receptors pinpoints the critical contribution of this structural feature in BoNT/B, DC, and G for the membrane binding. Thermodynamic binding analysis deciphers that the insertion of the H_C_ loop into the lipid bilayer compensates for the large entropic penalty imposed by the dual-receptor binding. Our results show that the hydrophobic H_C_ loop of BoNT/B, DC, and G is an integral component of the receptor binding and that ternary interactions between three different classes of molecules—proteins, gangliosides and lipids—are needed to mediate stable and high-affinity binding of BoNT/B, DC, and G to exert their exquisite toxicity.

## Results

### An exposed hydrophobic loop resides between the ganglioside- and protein receptor-binding sites of BoNT/B, DC, and G

Crystal structures of the cell-binding domains of BoNT/B, DC and G (H_C_B, H_C_DC and H_C_G, respectively) reveal the presence of a flexible, exposed peptide loop interjacent to the conserved ganglioside- and Syt protein receptor-binding sites in the C-terminal half of the H_C_ domain (H_CC_)[22, 24, 40]. These H_C_ loops comprise amino acids E1245-E1252 in H_C_B, F1245-H1255 in H_C_DC and K1250-D1257 in H_C_G and are rich in aliphatic and especially aromatic amino acids (Fig 1A, S1 Fig). Interestingly, an analogous loop is absent in crystal structures of H_C_A, H_C_E and H_C_F which all employ SV2 as protein receptor [41–43]. To analyse the role of the H_C_ loop of BoNT/B, DC and G in the binding mechanism three mutants lacking 3–5 mainly aliphatic and aromatic amino acid residues were constructed (ΔH_C_ loop mutants: H_C_B ΔG1247-F1250, H_C_DC ΔY1251-F1253 and H_C_G ΔY1252-W1256). Subtype BoNT/B4, the most diverse BoNT/B subtype and major cause for food borne botulism e.g. in UK, displays a basic (Arg) instead of an aromatic residue (Phe) in the H_C_ loop (S1 Fig). Therefore, an additional H_C_B mutant was constructed comprising the exchanges I1248L/V1249L/F1250R based on the most diverse H_C_ loop of the BoNT/B4 subtype (S1 Fig) produced by the non-proteolytic strain Templin [44]. All full-length BoNT and H_C_ fragment ΔH_C_ loop mutants were expressed and isolated in yields similar to the corresponding wild-type constructs indicating no major structural impairment due to the absence of the H_C_ loop peptide. All ΔH_C_ loop mutants displayed a slightly faster migration pattern than the respective wild-type proteins in sodium dodecyl sulfate polyacrylamide gel electrophoresis (SDS-PAGE) analysis due to their decreased molecular weight (S1 Fig). Circular dichroism (CD)-spectroscopy and thermal denaturation experiments of the three ΔH_C_ loop mutants vs. the three wild-type H_C_ fragments indicated no change in secondary structure (S1 Fig).

### The hydrophobic H_C_ loop in between the ganglioside- and protein receptor-binding site is required for high toxicity

First, the potency of the three full-length BoNT ΔH_C_ loop mutants was assessed using the mouse phrenic nerve hemidiaphragm (MPN) assay. This *ex vivo* assay mimics the respiratory failure terminally induced in botulism by intoxicating an explanted hemidiaphragm [12, 30, 45]. The addition of BoNTs to the organ bath impairs nerve—muscle transmission and causes progressive muscle neuroparalysis. Dose—response calibration curves for BoNT/B, DC, and G wild-type toxins were generated and used to calculate the potency of the respective BoNT ΔH_C_ loop mutants. While 2 nM BoNT/B wild-type caused 50% paralysis in 78 min, 20 nM BoNT/B ΔG1247-F1250 were required for a similar paralysis time of 81 min which constitutes a residual potency of 0.83% (Fig 1B). Likewise, the potencies of BoNT/DC ΔY1251-F1253 and BoNT/G ΔY1252-W1256 were calculated as 0.40% and 0.16% of the respective BoNT wild-types. Hence, deletion of the H_C_ loop drastically impairs the neurotoxicity of BoNT/B, DC and G, indicating an integral role of this structural feature in their mechanism of action.

### ΔH_C_ loop mutants display reduced binding to dual-receptor Triton-micelles

Glutathione S-transferase (GST) pull down assays were set out to determine the contribution of the hydrophobic H_C_ loop to the toxin—receptor interactions. As previously demonstrated [12], H_C_B and H_C_G wild-type showed robust binding to their protein receptor Syt-II lacking the transmembrane domain (TMD; GST-rSyt-II 1–61), but reduced binding when Syt-II comprises its TMD (GST-rSyt-II 1–90) solvated in Triton X-100 micelles (Fig 1C, S2 Fig). Upon addition of complex polysialogangliosides, H_C_B and H_C_G wild-type reached their maximum binding to GST-rSyt-II 1–90 due to the dual-receptor interaction. Also BoNT/DC recognizes Syt-I and Syt-II as protein receptor [22, 26]. In GST pull down assays, H_C_DC wild-type shows a similar binding pattern to the diverse Syt-II configurations although at lower affinity, presumably due to the different Syt-binding site in the H_CC_ (Fig 1C). Gangliosides marginally contribute to H_C_DC-binding to Syt-II inserted into detergent micelles, indicating a lower affinity of ganglioside binding in BoNT/DC.

Analysis of pure protein-protein interactions between ΔH_C_ loop mutants (H_C_B ΔG1247-F1250, H_C_B I1248L/V1249L/F1250R, H_C_DC ΔY1251-F1253, H_C_G ΔY1252-W1256) and their protein receptor GST-rSyt-II 1–61 revealed binding affinities virtually identical to those of H_C_ wild-types clearly demonstrating that shortening the respective H_C_ loop did not impair protein receptor recognition (Fig 1C). Addition of the Syt-II TMD to insert GST-Syt-II 1–90 into Triton X-100 micelles almost abolished binding of H_C_B ΔG1247-F1250 as well as H_C_G ΔY1252-W1256 and drastically reduced binding of H_C_B I1248L/V1249L/F1250R while the already low affinity of H_C_DC was hardly reduced by the deletion ΔY1251-F1253. Addition of gangliosides did not restore binding of H_C_DC ΔY1251-F1253 and H_C_G ΔY1252-W1256, but partially rescued binding of H_C_B ΔG1247-F1250 to GST-rSyt-II 1–90 and fully restored the binding of H_C_B I1248L/V1249L/F1250R towards GST-rSyt-II 1–90. Hence, the H_C_ loop plays an integral role for BoNT/B, DC, and G in the recognition of membrane-embedded receptor structures. Furthermore, results of the B4-like H_C_ loop mutant indicate that aromatic residues are not an absolute requirement for membrane interaction.

### Kinetic analysis employing nanodisc-embedded receptor molecules reveals that interactions with gangliosides, proteins, and lipids are crucial for high-affinity binding

**Fig 2:**
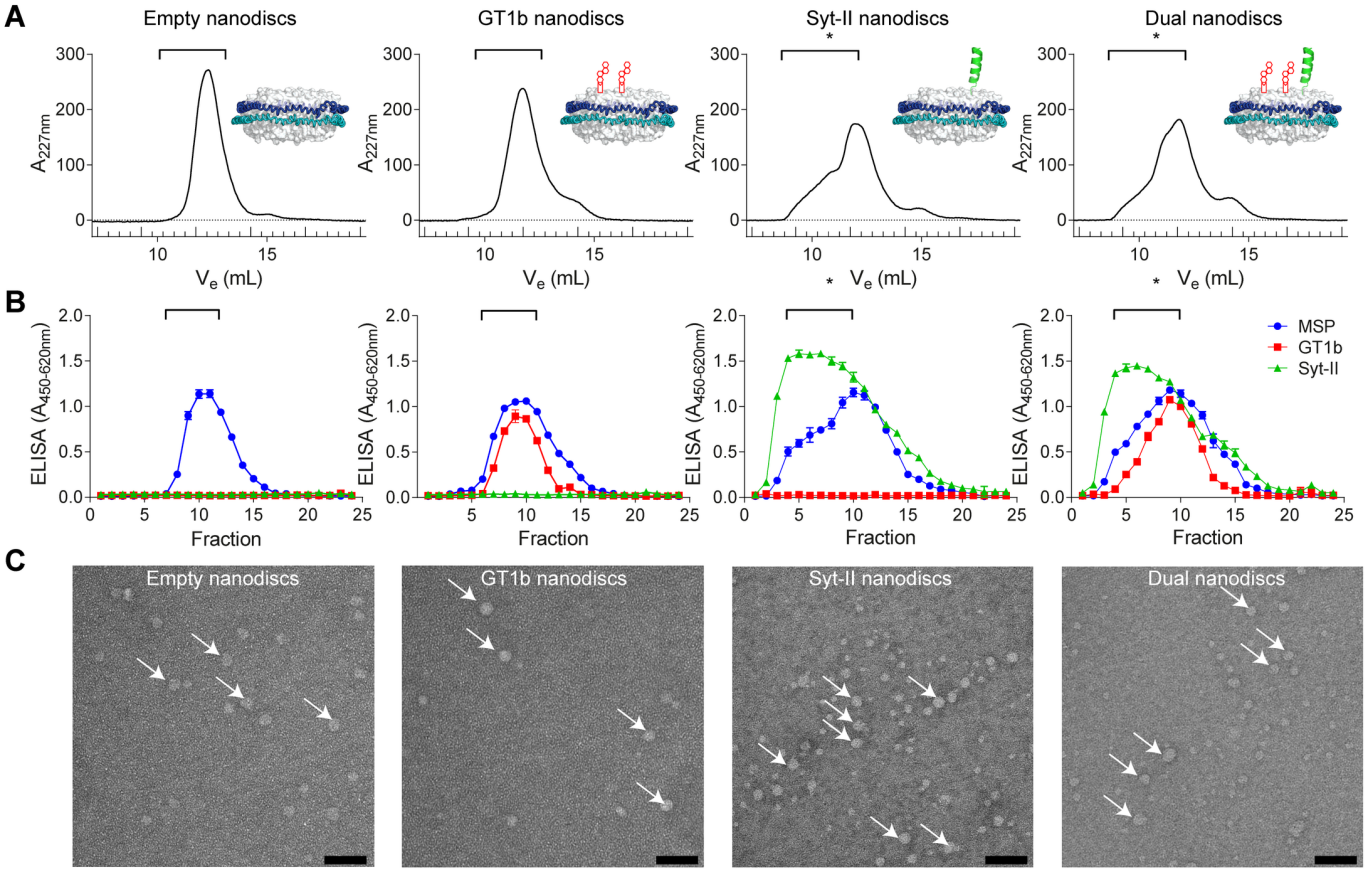
Generation and characterization of phospholipid-bilayer nanodiscs containing receptor molecules. **A** Size exclusion chromatography (SEC) elution profiles (absorption at 227 nm over elution volume V_e_) for nanodiscs containing either no receptor molecules (empty), GT1b only, GST-Syt-II 1–90 only, or GT1b and GST-Syt-II 1–90 (dual-receptor nanodiscs; schematic inserts modified from [73]). **B** ELISA results for the detection of membrane scaffold protein (MSP; via His-tag), GT1b, and Syt-II in the SEC-fractions. Brackets embrace the fractions used for the SPR measurements or further purification using the GST-tag on Syt-II (marked by asterisk). **C** Electron microscopy of the assembled nanodiscs purified by SEC (empty and GT1b nanodiscs) or SEC and batch-purification via the GST-tag (Syt-II nanodiscs and dual-receptor nanodiscs). Arrows indicate assembled discoidal nanodiscs of the correct diameter (13 nm) from top view. Scale bar = 50 nm.

To precisely decipher the contribution of the individual components of the receptor complex in a quasi-natural environment, we analyzed the binding of the recombinant H_C_ domains to receptor molecules embedded in phospholipid-bilayer nanodiscs (reviewed in [46]) by SPR measurements. Here, we generated four different types of nanodiscs harboring specific BoNT receptor components: (1) empty nanodiscs consisting only of membrane scaffold proteins (MSPs) and POPC lipids, (2) nanodiscs additionally containing GT1b as ganglioside receptors, (3) nanodiscs alternatively containing GST-rSyt-II 1–90 as protein receptor, and finally (4) nanodiscs containing both GT1b and Syt-II to analyze the dual-receptor binding (Fig 2).

**Fig 3:**
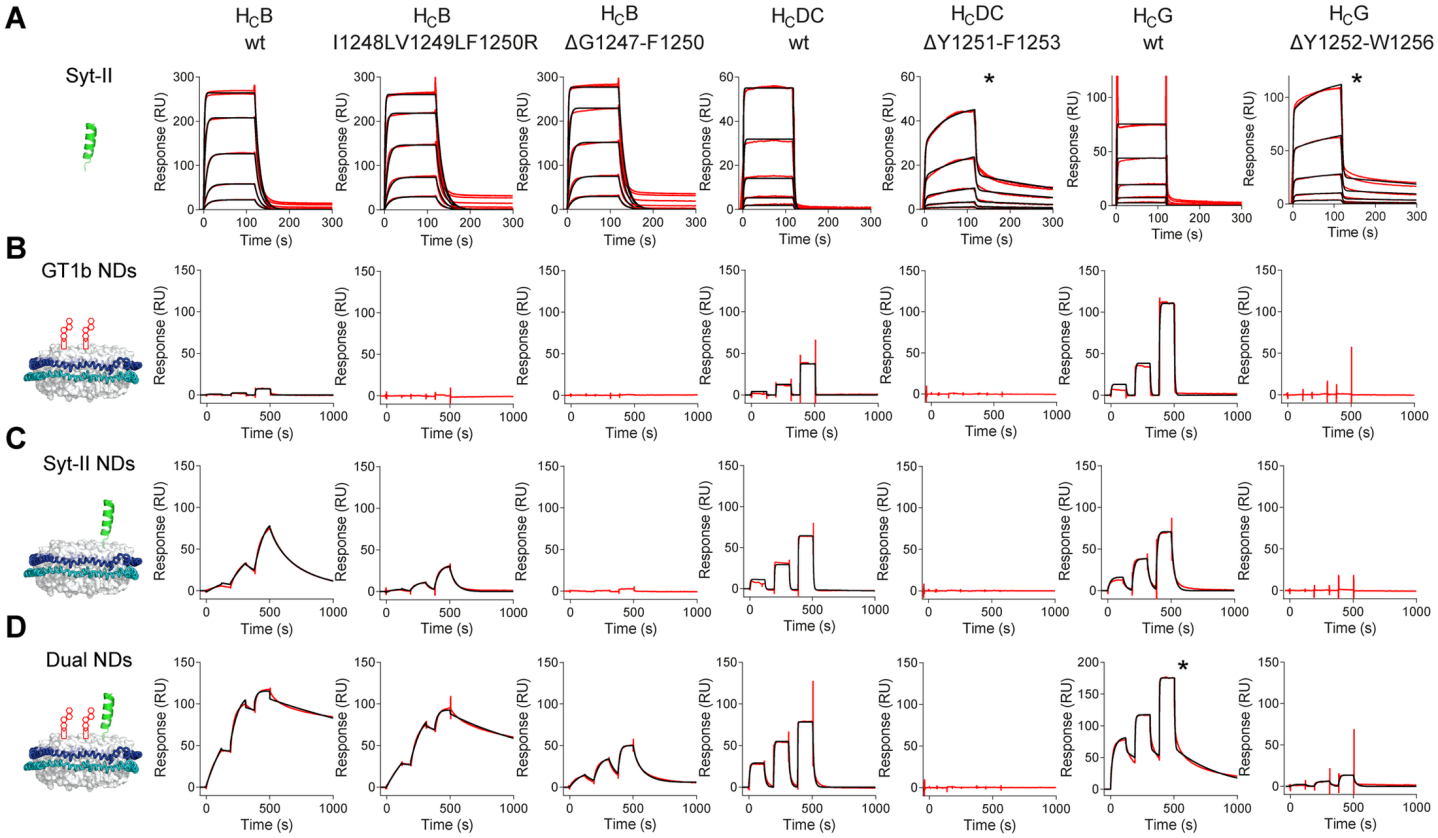
Kinetic analysis employing nanodisc-embedded receptor molecules reveals that interactions with gangliosides, proteins, and lipids are crucial for high-affinity binding. SPR-sensorgrams for the determination of binding kinetics of recombinant H_C_ receptor-binding domains to isolated GST-Syt-II 1–61 (**A**), GT1b nanodiscs (**B**), GST-Syt-II 1–90 nanodiscs (**C**), or dual-receptor nanodiscs containing both GT1b and GST-Syt-II 1–90 (**D**). Shown is one representative of two measured binding responses (red) overlaid with fits (black lines) from 1:1 Langmuir interaction models or a heterogeneous binding model (marked by asterisk), see Table 1 for kinetic data.

We determined the binding kinetics and affinity of the recombinant receptor-binding domains H_C_ of BoNT/B, DC, and G by SPR. First, we measured the binding of H_C_ to the intraluminal domain 1–61 of Syt-II to exclude detrimental effects by ΔH_C_ loop mutations on the Syt-binding site (Fig 3A). No differences were observed between the binding affinities of the H_C_ wild-types compared to the mutants tested (Table 1, S4 Fig), again demonstrating that the mutations did not impair binding to isolated protein receptor Syt-II. All binding kinetics show rapid association and immediate dissociation indicating that Syt-binding alone is insufficient to mediate high-affinity and stable receptor binding.

**Table 1:** Binding kinetics and affinity of the recombinant receptor-binding domains H_C_ and the respective H_C_ loop mutants towards different ligands (mean ± standard deviation of n = 2 replicate measurements; 25°C).

Ligand	Analyte	*k*_a_ (M^-1^s^-1^)^a^^,^^b^	*k*_d_ (s^-1^)^a^^,^^b^	*K*_D_ (M)^a^
**GST-rSyt-II 1–61**	H_C_B	5.7 ±1.8 × 10^5^	8.4 ±0.8 × 10^−2^	1.5 ±0.4 × 10^−7^
	H_C_B ΔG1247-F1250	5.1 ±0.9 × 10^5^	6.1 ±0.6 × 10^−2^	1.2 ±1.1 × 10^−7^
	H_C_B I1248L/V1249L/F1250R	5.5 ±0.8 × 10^5^	6.5 ±0.9 × 10^−2^	1.2 ±0.0 × 10^−7^
	H_C_DC^c^	2.3 × 10^5^	2.4 × 10^−1^	1.8 ±0.2 × 10^−6^
	H_C_DC ΔY1251-F1253^c^^,^^e^	1.9 × 10^5^	2.3 × 10^−1^	1.3 ±0.6 × 10^−6^
	H_C_G^c^	5.6 × 10^5^	4.1 × 10^−1^	9.0 ±2.2 × 10^−7^
	H_C_G ΔY1252-W1156^c^^,^^e^	3.7 × 10^5^	3.1 × 10^−1^	1.5 ±0.6 × 10^−6^
**Nanodiscs**	H_C_B^d^	5.9 ±4.7 × 10^4^	1.2 ±0.4 × 10^0^	3.2 ±3.2 × 10^−5^
**+ GT1b**	H_C_B ΔG1247-F1250	*n*.*b*.	*n*.*b*.	*n*.*b*.
	H_C_B I1248L/V1249L/F1250R	*n*.*b*.	*n*.*b*.	*n*.*b*.
	H_C_DC	2.0 ±1.5 × 10^5^	2.3 ±0.2 × 10^0^	1.5 ±1.1 × 10^−5^
	H_C_DC ΔY1251-F1253	*n*.*b*.	*n*.*b*.	*n*.*b*.
	H_C_G	2.5 ±1.2 × 10^5^	1.2 ±0.4 × 10^0^	5.8 ±4.4 × 10^−6^
	H_C_G ΔY1252-W1156	*n*.*b*.	*n*.*b*.	*n*.*b*.
**Nanodiscs**	H_C_B	2.0 ±1.8 × 10^5^	4.0 ±3.2 × 10^−2^	2.1 ±0.2 × 10^−7^
**+ GST-rSyt-II 1–90**	H_C_B ΔG1247-F1250	*n*.*b*.	*n*.*b*.	*n*.*b*.
	H_C_B I1248L/V1249L/F1250R	9.4 ±0.8 × 10^3^	2.9 ±0.9 × 10^−2^	3.2 ±1.3 × 10^−6^
	H_C_DC	5.6 ±2.9 × 10^5^	4.9 ±2.5 × 10^−1^	8.7 ±0.1 × 10^−7^
	H_C_DC ΔY1251-F1253	*n*.*b*.	*n*.*b*.	*n*.*b*.
	H_C_G	2.4 ±2.0 × 10^5^	1.1 ±0.9 × 10^−1^	4.4 ±0.0 × 10^−7^
	H_C_G ΔY1252-W1156	*n*.*b*.	*n*.*b*.	*n*.*b*
**Nanodiscs**	H_C_B	8.6 ±0.4 × 10^4^	5.0 ±0.0 × 10^−4^	5.9 ±0.2 × 10^−9^
**+ GT1b**	H_C_B ΔG1247-F1250	2.7 ±1.2 × 10^5^	6.4 ±2.3 × 10^−2^	2.4 ±0.2 × 10^−7^
**+ GST-rSyt-II 1–90**	H_C_B I1248L/V1249L/F1250R	8.1 ±0.0 × 10^4^	9.5 ±0.1 × 10^−4^	1.2 ±0.0 × 10^−8^
	H_C_DC	3.9 ±0.4 × 10^6^	6.3 ±0.6 × 10^−1^	1.6 ±0.0 × 10^−7^
	H_C_DC ΔY1251-F1253	*n*.*b*.	*n*.*b*.	*n*.*b*.
	H_C_G	3.2 ±2.9 × 10^6^	1.5 ±1.7 × 10^−1^	3.8 ±2.0 × 10^−8^
	H_C_G^e^	3.7 ±0.3 × 10^6^	2.2 ±0.2 × 10^−1^	6.0 ±1.0 × 10^−7^
	H_C_G^f^	4.9 ±0.3 × 10^6^	2.6 ±0.0 × 10^−3^	5.4 ±0.3 × 10^−9^
	H_C_G ΔY1252-W1256	*n*.*a*.	*n*.*a*.	1.1 ±0.0 × 10^−6^

^a^ *n*.*b*.: no binding observed

^b^ *n*.*a*.: not analyzed

^c^ Reliable kinetic binding data (*k*_*a*_, *k*_*d*_) could only be obtained for one of the two replicates due to rapid binding kinetics.

^d^ Only very low binding levels observed.

^e^ Analyzed by heterogeneous ligand-binding model: low-affinity component.

^f^ Analyzed by heterogeneous ligand-binding model: high-affinity component.

Subsequently, we analyzed the binding of H_C_ to receptor molecules embedded into phospholipid-bilayer nanodiscs by SPR. Empty nanodiscs were immobilized on the negative control flow cells while either GT1b-, Syt-II- or dual-receptor nanodiscs were immobilized on the measurement flow cells using the His-tag fused to MSP. Hereby, we ensured that any additional binding signals could only be caused by receptor molecules integrated into nanodiscs.

H_C_ wild-types showed low binding affinities in the μM-range to GT1b nanodiscs while the ΔH_C_ loop mutants lacked any binding (Fig 3B, Table 1). This indicates that the H_C_ loop is needed for the interaction with GT1b integrated in lipid membranes, but this set-up does not differentiate whether the H_C_ loop mediates interactions with the ceramide portion of the gangliosides or the POPC lipids. However, when binding to Syt-II nanodiscs was tested (Fig 3C), the complete lack of binding of all ΔH_C_-loop mutants indicates an indispensable role of the H_C_ loop when BoNT/B, DC, and G bind to Syt-II embedded into a lipid bilayer. These results were also in good agreement with the pull-down data (Spearman r = -0.96; Fig 1C, S4 Fig). Interestingly, the H_C_B I1248L/V1249L/F1250R mutant mimicking the H_C_ loop of subtype BoNT/B4 showed binding to Syt-II nanodiscs albeit with ~15 times lower affinity than H_C_B wild-type. Of the eight residues (E1245-E1252) comprising the BoNT/B H_C_ loop, only three residues are strictly conserved and two aliphatic residues are similar in all eight BoNT/B subtypes (S1 Fig). Here, exchange of F1250R might be the main cause for the reduced affinity of H_C_B I1248L/V1249L/F1250R. Overall, H_C_ wild-type binding to Syt-II embedded into nanodiscs is more stable than the binding to Syt-II 1–61 only or to gangliosides embedded into nanodiscs, but not sufficiently stable to exert the exquisite potency observed.

Finally, when binding to dual-receptor nanodiscs was analyzed, high-affinity and stable interactions were only observed for H_C_B wild-type as well as H_C_B I1248L/V1249L/F1250R (Fig 3D), indicating that the H_C_ loop binding is conserved across the BoNT/B subtypes despite differences in amino acid sequence. This demonstrates that three components, the ganglioside-binding site, the protein receptor-binding site, and the hydrophobic H_C_ loop, are crucial for efficient membrane binding. Along this line, the mutant H_C_B ΔG1247-F1250 showed a 40-fold reduced affinity and unstable binding compared to H_C_B wild-type. An even more drastic situation accounts for BoNT/DC and G whose ΔH_C_ loop mutants showed no or only very low binding, which is in good agreement with results obtained by pull-down assays (Spearman r = -0.99; S4 Fig). The binding affinity of 5.9 ± 0.2 nM for H_C_B wild-type to dual-receptor nanodiscs corresponds well to previously reported *K*_D_s determined by pull down of BoNT/B by Syt-II and GT1b incorporated in Triton X-100 micelles of 7.0 ± 0.6 nM [23]. Binding of full-length BoNT/B to Syt-II and GT1b incorporated in lipid vesicles or exosomes resulted in slightly higher affinities of 0.23 nM when measured in a filtration assay [47], or 0.6 nM by SPR [48]. Latter deviations could be explained by different read-out systems and the more physiological membrane composition of the exosomes, respectively. The affinity of H_C_DC wild-type to dual-receptor nanodiscs is similar to an apparent *K*_D_ determined by pull-down assays using Triton X-100 micelles (160 nM vs. 172 ± 14 nM [26]). Altogether, the marked reduction in dissociation-rate constants due to the formation of a highly stable BoNT-receptors complex was clearly visible in our work. In conclusion, our data clearly show that the current dual-receptor model has to be extended by the H_C_ loop-mediated interaction of BoNT/B, DC, and G with the lipid membrane, which ensures the high-affinity and stable receptor binding to a tripartite-receptor binding model.

### Membrane integration of the H_C_ loop compensates for the entropic penalty inflicted by dual-receptor binding at physiological temperatures

**Fig 4:**
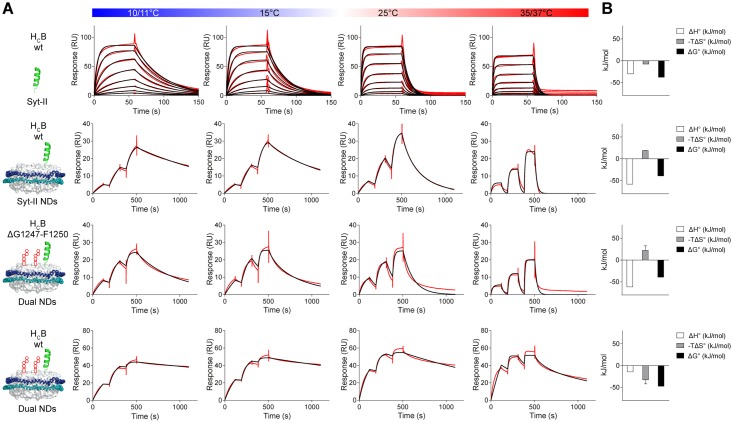
Membrane integration of the H_C_ loop compensates for the entropic penalty inflicted by dual-receptor binding at physiological temperatures. **A** Representative SPR-sensorgrams for determination of thermodynamic binding parameters for H_C_B wild-type or the ΔH_C_ loop mutant to isolated Syt-II, Syt-II nanodiscs, or dual-receptor nanodiscs measured at the temperatures indicated in the bar above the graphs. **B**. Thermodynamic profile depicting binding enthalpy (ΔH°), entropy (-TΔS°), and the standard Gibbs free energy (ΔG°) for those interactions; see also Table 2 for thermodynamic data.

For a deeper understanding of the underlying mechanism governing the contribution of the hydrophobic H_C_ loop to the high-affinity binding, we determined the thermodynamic binding parameters exemplarily for the H_C_B wild-type and ΔG1247-F1250 mutant to Syt-II or dual-receptor nanodiscs, respectively, by SPR. To this aim, the temperature-dependence of the binding affinity was determined by measuring the interaction at four different temperatures (11°C, 15°C, 25°C, and 37°C) from which ΔG°, ΔH°, and -TΔS° were calculated by van’t Hoff plots (Fig 4, S5 Fig).

The thermodynamic binding parameters determined for binding of H_C_B wild-type to isolated Syt-II by SPR were in close agreement with the binding parameters previously determined by isothermal titration calorimetry [24] and show that the interaction is favored by both entropy and enthalpy (Fig 4, Table 2). Binding of H_C_B wild-type to nanodisc-incorporated Syt-II was largely driven by enthalpy (-58.4 kJ/mol) but opposed by entropy (18.99 kJ/mol), indicating a different mode of binding despite similar free Gibbs energy (ΔG°). On the contrary, binding of H_C_B wild-type to dual-receptor nanodiscs was essentially driven by a gain in binding entropy (-32 kJ/mol) while the enthalpic contribution (ΔH° = -14 kJ/mol) was minor. The thermodynamic profile of the interaction of H_C_B ΔH_C_ loop with dual-receptor nanodiscs is similar to that of H_C_B wild-type with Syt-II-only nanodisc, exhibiting an important contribution of the H_C_ loop to the high gain in entropy. As a direct consequence, at physiological temperatures of 37°C, which reduces the contribution of entropy compared to 25°C (–TΔS°), only H_C_B wild-type still displays a high-affinity interaction with dual-receptor nanodiscs while the affinity of H_C_B ΔH_C_ loop is reduced ~50-fold (S1 Table). In general, our thermodynamic analysis elucidates that the interplay of all three receptor components is indispensable for effective stabilization of the tripartite toxin—receptor complex under physiological temperatures, with the H_C_ loop providing a significant gain in entropy.

**Table 2:** Thermodynamic binding parameters of H_C_B and H_C_B ΔG1247-F1250 (mean ± SD of n = 4 (H_C_B—Syt-II) or n = 2 (others) replicate measurements).

Interaction	ΔH°(kJ/mol)	–TΔS° (kJ/mol at 25°C)	ΔG° (kJ/mol at 25°C)	*K*_D_ (nM at 25°C)^b^
H_C_B—Syt-II^a^	-30.9	-11.2	-42.2	34
H_C_B—Syt-II	–30.3 ±0.7	–7.9 ±0.7	–38.27 ±0.02	190 ±15
H_C_B—Syt-II nanodiscs	–58.4 ±0.2	18.99 ±0.03	–39.4 ±0.2	140 ±14
H_C_B ΔG1247-F1250 –Dual-receptor nanodiscs	–61 ±10	22 ±10	–39.5 ±0.4	120 ±31
H_C_B—Dual-receptor nanodiscs	–14 ±10	–32 ±9	–47.8 ±0.1	4.4 ±0.3

^a^ For comparison: values taken from [24].

^b^ For comparison: affinity data at 25°C. Full kinetic and affinity data shown in supporting Table 1.

## Discussion

**Fig 5:**
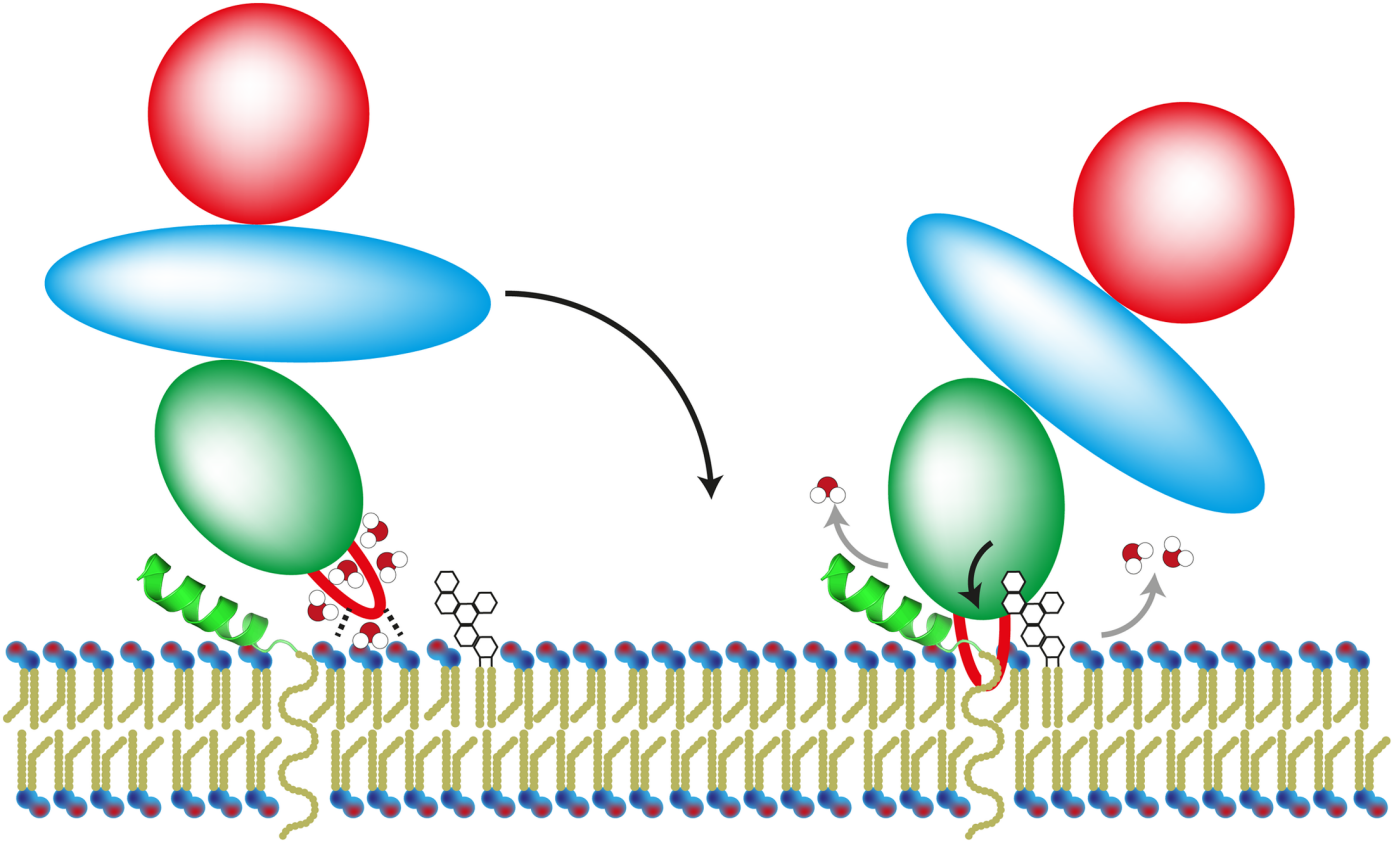
Functional model of ternary interactions of BoNT/B, DC, and G with the neuronal membrane. BoNT (H_C_, green oval; H_C_ loop, red) forms a highly stable BoNT-receptors complex, involving an SV protein (Syt-II, green helix), a carbohydrate portion (empty hexagons) of gangliosides, and membrane lipids, that is critical to confer the toxin’s exquisite toxicity. Initial electrostatic interactions of H_C_ loop residues with negatively charged phospholipid-headgroups cause a gain in binding enthalpy, which cannot overcome the unfavorable contribution of the binding entropy by restrictions on the rotational and translational degrees of freedom upon binding. Only ganglioside-triggered burial of the H_C_ loop into the membrane bilayer can compensate the initial entropic penalty by releasing water molecules (white-red balls) from the interaction surface.

In this manuscript, we analyzed for the first time the interaction of BoNT/B, DC, and G with their receptor components in the quasi-natural environment of phospholipid-bilayer nanodiscs which allowed new insights into the BoNT-receptor interaction and their kinetics at physiological conditions. We show how the hydrophobic H_C_ loop located between the ganglioside- and protein receptor-binding sites of the toxin plays a crucial role in the mechanism of action of BoNT/B, DC, and G, all binding the protein receptors Syt-I and Syt-II. Figuratively speaking, the toxin provides its H_C_ loop as anchor to hook up with the eukaryotic synaptic cell membrane (Fig 5). This H_C_ loop stabilizes high-affinity binding of BoNT/B, DC, and G to the membrane-embedded receptors, thereby contributing to their exquisite neurotoxicity. Moreover, our thermodynamic binding analysis exhibits that the H_C_ loop integrating into the membrane during receptor binding compensates for the loss of rotational freedom upon the dual-receptor binding. In essence, at least for BoNT/B, DC, and G, stable toxin—receptor interactions are based on ternary interactions involving proteins, gangliosides, and lipids (Fig 5) and not just on binary interactions as previously predicted [10].

Crystal data of the BoNT/B-Syt-II binary toxin—receptor complex suggested a close proximity of the H_C_ loop to the cell membrane upon protein—receptor binding, and a role of this hydrophobic loop in membrane interaction was hypothesized [23, 24]. This hypothesis was revived but not proven upon crystallization of H_C_G, also comprising a prominent H_C_ loop too flexible to be visible in the crystal structure [40]. Before structural data was available, site-directed mutagenesis of the W1258/Y1259 (WY) motif in BoNT/C H_C_ showed a moderate effect on ganglioside binding and strong effects on synaptosomal membrane binding and neurotoxicity [30, 49]. Consecutive structural data of H_C_C allocated this WY motif into an analogous H_C_ loop and confirmed the previous binding and toxicity data [34, 35]. In parallel, Strotmeier et al. could demonstrate experimentally the importance of an analogous hydrophobic H_C_ loop (F1240-Y1246) observed in the BoNT/D H_C_ crystal structure for neuronal membrane binding and neurotoxicity [50], which was essentially confirmed afterwards [51]. The H_C_ fragment of BoNT/DC, 74% identical to BoNT/C H_C_, employs Syt-I and -II as protein receptor and displays an extended H_C_ loop. The mutation W1252A in the loop region reduced binding of H_C_DC to coated ganglioside GM1 as well as to primary cortical neurons, which led the authors to term the region ganglioside-binding loop (GBL) [35]. Independent mutational analysis showed drastically reduced binding of H_C_DC H_C_ loop mutants to P19 neurons as well as to ganglioside-containing POPC liposomes, irrespective of the carbohydrate moiety of gangliosides GM1a, GD1a, and GQ1b used in these SPR analyses [38]. Due to the absence of H_C_DC binding to POPC-only liposomes, Nuemket et al. excluded any lipid membrane interaction of the H_C_ loop, but postulated instead that the hydrophilic portion of the ceramide would be targeted by the H_C_ loop region [38]. However, the highest concentration of the H_C_-fragment tested was 500 nM; thus highly transient and low-affinity interactions frequently observed for lipid-binding proteins [52, 53] remain undetected by the SPR method. Very recently the work by Zhang et al. added significantly to our understanding of the ganglioside recognition as well as the membrane interaction by the H_C_ loop region for BoNT/DC. Their most intriguing finding was that the H_C_ loop of BoNT/DC directly interacts with lipids only. In addition, the three single mutations Y1251A, W1252A, and F1253A designed by Nuemket et al. abolished H_C_DC interaction with lipids as well as liposome-embedded gangliosides [39]. These data agree well with our results showing that ΔH_C_ loop mutants do not bind to GT1b embedded in nanodiscs (Fig 3B). Altogether, the above data for BoNT/C, D, and DC cannot decipher whether the H_C_ loop directly participates in ganglioside binding or membrane binding only or both, because isolated gangliosides already form micelles by themselves due to their ceramide portion, which enables insertion of the H_C_ loop into the ganglioside micelle membrane. For BoNT/B, biochemical and structural studies definitely demonstrated that the carbohydrate moiety of only a single ganglioside exclusively interacts with residues of the conserved ganglioside-binding site in the neighborhood of the H_C_ loop but not with H_C_ loop residues themselves [11, 54, 55]. Analogous conclusions can be drawn for BoNT/G [12, 28, 40]. So far, no structural study has visualized or pointed towards direct interaction between an H_C_ loop and the carbohydrate moiety of gangliosides. Our experimental approach, using only the luminal domain of the protein receptor Syt-II in the absence of any lipids and only Syt-II integrated either into Triton X-100 micelles or nanodiscs, unambiguously proves an interaction of the H_C_ loop with the lipids. Here, deleting terminal residues at the tip of the H_C_ loop did not impair the interaction of H_C_B, H_C_DC, and H_C_G with isolated Syt-II, but caused a strong reduction of the binding affinity towards nanodisc- or Triton X-100 micelle-embedded Syt-II. These findings can only be explained by interactions of the H_C_ loop with membrane lipids. Along this line, Zhang et al. were able to demonstrate low-affinity binding of H_C_DC wild-type, but not H_C_C and H_C_D, to pure PC liposomes by immunoblot analysis which was abolished by above-mentioned single-residue mutations in the H_C_ loop [39].

Remarkably, BoNT/B, DC, and G ΔH_C_ loop mutants showed a strong reduction in binding and toxicity compared to the respective BoNT wild-type (Fig 1B), clearly demonstrating an integral contribution of the H_C_ loop to the binding, uptake, and putative membrane insertion of H_N_ for LC translocation into the cytosol. A mechanistic explanation of how the H_C_ loop mediates the high-affinity interaction was evolved by measuring the binding thermodynamics of the H_C_B interaction. Here, despite similar overall binding affinities of H_C_B to either isolated Syt-II or Syt-II integrated into nanodiscs, different contributions of the binding enthalpy and binding entropy were calculated. Although the much larger binding enthalpy for the binding of H_C_B to membrane integrated Syt-II leads to additional electrostatic interactions between positive protein surface charges and negatively charged phospholipid headgroups [56], a large unfavorable contribution of the binding entropy indicates strong restrictions on the rotational and translational degrees of freedom upon binding [57]. The binding of H_C_B ΔH_C_ loop mutant to dual-receptor nanodiscs revealed the same thermodynamic reaction profile. In contrast, a large and favorable binding entropy, which is typically associated with the burial of hydrophobic groups away from the solvent [58], was measured for H_C_B wild-type bound to dual-receptor nanodiscs, which indicates an integration of the H_C_ loop into the membrane bilayer. Indeed, the BoNT/B H_C_ loop is solvated by up to 20 molecules of water [55] which are released upon membrane integration of the H_C_ loop. It is noteworthy that this large gain in binding entropy is only observed for the dual-receptor nanodiscs, implying that the interaction with the carbohydrate portion of GT1b is needed to trigger the membrane insertion of the H_C_ loop (Fig 4B). This result is in agreement with recent findings that binding to the carbohydrate portion of gangliosides confers specificity, whereas binding strength is mediated by additional interactions of the toxins with the neuronal membrane itself [59]. Hence, the sequence of the tripartite interactions is likely to be i) specific adherence of BoNT/B to a ganglioside, ii) integration of BoNT/B H_C_ loop into the membrane stabilizing the BoNT-receptor complex and iii) binding of BoNT/B to synaptotagmin to direct its uptake into an SV to act locally in the synapse. However, the almost identical binding affinities between H_C_B wild-type bound to Syt-II nanodiscs and H_C_B ΔH_C_ loop-mutant bound to dual-receptor nanodiscs imply that the contribution of the ganglioside to the binding strength equals that of the H_C_ loop membrane insertion. Nevertheless, binding of H_C_B wild-type to empty nanodiscs was not reliably measurable by SPR, which points towards a very low intrinsic affinity of the H_C_ loop for isolated lipid membranes, as previously observed also by Zhang et al. and Nuemket et al. [38, 39]. Despite the ubiquitous abundance of lipid membranes in the organism, the very low intrinsic affinity of the H_C_ loop prevents off-target binding which would effectively lower the neurotoxicity.

Beside BoNT/B, DC, and G employing Syt as protein receptor, also BoNT/C, to which no protein receptor has been ascribed yet, displays an exposed H_C_ loop [34]. This suggests that an H_C_ loop—membrane insertion can occur also by BoNT serotypes not employing Syt as protein receptor. In contrast, in BoNT/A the H_C_ loop is crippled and completely absent in the related BoNT/E and F. The absence of an analogous H_C_ loop—membrane interaction of BoNT/A and E could also be compensated by the additional binding to the conserved N-glycan in SV2A-C [18, 19, 21]. Also other mechanisms can contribute to the high-affinity interaction with the membrane. For instance, BoNT/A interactions with the lipid membrane have been allocated to the N-terminal subdomain of the H_C_-fragment [61].

Finally, our results show that direct interactions between BoNT/B, DC, and G and the membrane already take place at neutral pH during the initial binding step. It has been shown before that interactions with GT1b are needed for the formation of a translocation pore at low pH [62]. The integration of the hydrophobic H_C_ loop in the membrane potentially primes BoNT/B, DC, and G for subsequent conformational changes needed to insert the translocation domain H_N_ into the membrane.

The H_C_ loop is always exposed and of sufficient length to constitute an epitope for a monoclonal antibody, thereby representing a novel target structure to neutralize BoNT/B, DC, and G in a clinical setting. MPN hemidiaphragm data clearly illustrate the loss of potency upon H_C_ loop deletion, which is comparable to blockade by an antibody [63].

Interestingly, membrane interaction of a hydrophobic loop for high-affinity binding is a common feature that is shared between BoNT/B, DC, G, and the broadly neutralizing anti-HIV-1 antibody 4E10 [64, 65]. 4E10 binds to contiguous epitopes within the membrane proximal external region (MPER) of the envelope transmembrane glycoprotein gp41 as well as host membrane lipids to exert its neutralizing activity.

In conclusion, our data show how membrane interactions of BoNT/B, DC, and G via a hydrophobic H_C_ loop contribute to the formation of a highly stable BoNT-receptors complex, involving a protein, gangliosides, and lipids, that is critical to confer the toxin’s exquisite toxicity (Fig 5).

## Materials and methods

### Ethics statement

All experiments were performed in accordance with the German Tierschutzgesetz (TierSchG, 29^th^ March 2017) and Tierschutz-Versuchstierverordnung (TierSchVersV, 1^st^ August 2013) and with the guidelines established by the European Community Council Directive n° 2010/63/EU and approved by the local authority veterinary services (Veterinäramt Hannover, protocol file number §4/019).

### Reagents

Recombinant MSP 1E3D1, sodium cholate hydrate, octyl β-D-glucopyranoside (OG), POPC, anhydrous chloroform, trisialoganglioside GT1b from bovine brain, GD1a, and rat brain extract were obtained from Sigma Aldrich, GM1 and GD1b were obtained from Calbiochem while GM3 was from Avanti Polar Lipids. MSP was dissolved in phosphate buffered saline (PBS, pH 7.3) containing 4 mM OG, the concentration was quantified photospectrometrically using a molecular extinction coefficient of ε_280_ = 29,400 M^-1^cm^-1^ and stored at -20°C. POPC was dissolved in chloroform while all gangliosides were dissolved in PBS and stored at -20°C. Bio-Beads SM-2 were obtained from Bio-Rad, activated in methanol (Merck), and stored in distilled sterile water at 4°C until used. Protease-free bovine serum albumin (BSA, fraction V), skimmed milk powder, and reduced glutathione were obtained from Carl Roth while a PageRuler prestained protein ladder was obtained from Thermo Fisher Scientific. Immobilon P PVDF-membranes (0.45 μm) for western blotting were from Merck Millipore while BioTrace NT nitrocellulose membranes (0.2 μm) for dot blots were from Pall corporation.

### Antibodies

A monoclonal mouse anti 6×His epitope tag antibody (clone His.H8) was obtained from Thermo Fisher Scientific (Pierce) while an affinity-purified polyclonal rabbit antibody targeting amino acids 1 to 11 from mouse synaptotagmin 2 was obtained from Synaptic Systems. A mouse monoclonal antibody targeting GT1b ganglioside (clone GT1b-2b) was obtained from Merck Millipore. All primary antibodies were tested for specific detection of their respective target by western and dot blot and showed no cross-reactivity at the used concentrations (S6 Fig). Horseradish peroxidase (HRP)-coupled goat anti-mouse IgG (H+L) or anti-rabbit IgG (H+L)-specific antibodies were obtained from Dianova or KPL.

### Plasmid construction

Plasmids encoding the H_C_-fragment of BoNT/B and G fused to a C-terminal Streptag (H_C_BS, H_C_GS), full-length BoNT/B and G equipped with a C-terminal Streptag (scBoNTBSL, scBoNTGS), as well as GST fusion proteins of rSyt-II 1–61 and rSyt-II 1–90 have been described previously [12, 27, 54]. The plasmids pH_C_DCS and pH6tBoNTDCS, encoding the H_C_-fragment of BoNT/DC fused to a C-terminal Streptag (H_C_DCS; AA 863–1285) and full-length BoNT/DC equipped with an N-terminal His6tag and a C-terminal Streptag (H6tBoNTDCS), respectively, were generated by amplifying the corresponding ORFs using genomic DNA of *C*. *botulinum* strain OFD05 (Gen ID AB461915; kind gift from Keiji Nakamura, Osaka Prefecture University, JP) as template and cloned into modified pQe3 vectors cut with BamH I/Xma I. The plasmid pH_C_BS I1248L/V1249L/F1250R contains the most diverse H_C_ loop of the BoNT/B4 subtype produced by the non-proteolytic Group II strain Templin [44] which was isolated from a home-made sheep’s ham associated with food-borne botulism in Germany, 2006. Sequence of the BoNT/B4 (GenBank number: MG545727) showed 99.1% identity to the prototype BoNT/B4 sequence (Genbank number: ABM73987) from strain Ecklund 17B [6] at amino acid level.

The ΔH_C_ loop (deletion) mutants pH_C_BS ΔG1247-F1250, pH_C_BS I1248L/V1249L/F1250R, pBoNTBSL ΔG1247-F1250, pH_C_DCS ΔY1251-F1253, pH6tBoNTDCS ΔY1251-F1253, pH_C_GS ΔY1252-W1256, and pBoNTGS ΔY1252-W1256 were generated by PCR, applying the GeneTailor site-directed mutagenesis system (Life Technologies, Darmstadt, Germany) and suitable primers (Eurofins, Ebersberg, Germany). Nucleotide sequences of all newly generated constructs were verified by DNA sequencing (GATC Biotech, Konstanz, Germany).

### Expression and purification of recombinant proteins

In general, expression and purification of active full-length BoNT and mutants thereof were conducted under biosafety level 2 containment (project number GAA A/Z 40654/3/123). The isolation of HA33, GST-rSyt-II 1–61, GST-rSyt-II 1–90, wild-type H_C_BS, single-chain (sc) scBoNTBSL, HcGS, and scBoNTGS have been described previously [12, 27, 54, 66]. H_C_DCS and H6tBoNTDCS variants were expressed analogously. After isolation via C-terminal StrepTag according to the manufacturer’s instruction (StrepTactin resin; IBA GmbH, Göttingen, Germany), H_C_DCS wild-type and H_C_DCS ΔY1251-F1253 were purified further by size exclusion chromatography (Superdex 75, GE Healthcare, Freiburg, Germany) in PBS, pH 7.4. H6tBoNTDCS wild-type and H6tBoNTDCS ΔY1251-F1253 were first isolated by IMAC (Co^2+^-Talon matrix; Takara Bio Europe S.A.S., Saint-Germain-en-Laye, France) and subsequently by affinity chromatography employing StrepTactin resin in 100 mM Tris-HCl, pH 8.0. GST fusion proteins eluted by glutathione were dialyzed against PBS, pH 7.4, two times with and two times without β-mercaptoethanol. Desired protein fractions were pooled, frozen in liquid nitrogen, and kept at −70°C. For CD analysis, desired volume of H_C_ proteins was dialyzed against 100 mM Tris-HCl, pH 8.0, 150 mM NaCl. Protein concentrations were determined subsequent to SDS-PAGE and Coomassie blue staining by using an LAS-3000 imaging system (Fuji Photo Film), the AIDA 3.51 software (Raytest, Straubenhardt, Germany), and BSA (100–1600 ng) as reference protein.

### Mouse phrenic nerve hemidiaphragm assay

The MPN assay was performed as described previously [13, 19]. Mice of strain RjHan:NMRI (18–25 g, Janvier, St Berthevin Cedex, France) were sacrificed by trained personnel before dissection of organs. First, mice were euthanized by CO_2_ anesthesia and subsequently exsanguinated via an incision of the ventral aspect of the throat. Then the chest of the cadaver was opened. To limit the consumption of mice, the left and right phrenic nerve hemidiaphragms were excised from female mice and placed in an organ bath containing 4 ml of Earle’s Balanced Salt Solution. The pH was adjusted to 7.4 and oxygen saturation was achieved by gassing with 95% O_2_ and 5% CO_2_. The phrenic nerve was continuously electro-stimulated at a frequency of 1 Hz with a pulse duration of 0.1 ms and a current of 25 mA to achieve maximal contraction amplitudes. Isometric contractions were recorded with a force transducer (Scaime, Annemasse, France) and the software VitroDat (Föhr Medical Instruments GmbH (FMI), Seeheim, Germany). The resting tension of the hemidiaphragm was approximately 10 mN. In each experiment, the preparation was first allowed to equilibrate for 15 min under control conditions. Then, the buffer was exchanged to 4 ml of Earle’s Balanced Salt Solution supplemented with 0.1% BSA and varying dilutions of wild-type BoNT/B, BoNT/DC, and BoNT/G. The times required to decrease the amplitude by 50% (paralysis time t½ ≤ 180 min) for three or four BoNT concentrations (each n ≥ 3) were used to construct the calibration curves for scBoNT wild-type to which logarithmic or power functions were fitted (y (scBoNTBSL; 650/2000/6500 pM) = -19.84ln(x) + 227.3, R^2^ = 0.9999; y (scH6tBoNTDCS; 100/300/1000 pM) = -13.49ln(x) + 146.28, R^2^ = 0.9978; (y (BoNT/G; 0.6, 2.0, 6.0 and 20 nM) = 97.123x^-0.271^; R^2^ = 0.9967). These functions were used to convert the mean paralysis times t_½_ determined for 200 nM scBoNTBSL ΔG1247-F1250 (n = 4), 30 nM H6tBoNTDCS ΔY1251-F1253 (n = 4), and 60 nM BoNTGS ΔY1252-W1256 (n = 2) into the corresponding scBoNT wild-type concentrations and to express them as relative biological activity.

### Circular dichroism analysis

Circular dichroism (CD) data was collected with a Jasco J-810 spectropolarimeter in a 1-mm path length cuvette with a concentration of 10 μM H_C_BS or 3 μM H_C_GS/H_C_DCS degassed. Spectra were recorded at 22°C from 195 to 250 nm with 100 nm/min, response of 1 s, standard sensitivity, bandwidth of 1 nm, and five accumulations. Spectra were analyzed, processed, and visualized using Spectra Manager II software (JASCO International Co. Ltd., Tokyo, Japan). Subsequent temperature-induced denaturation was performed by monitoring the CD signal at 210 nm from 25°C to 70°C with a stepwise temperature increase of 2.5°C every 6 min.

### GST pull-down assays

The GST pull-down assays were similarly performed as previously described [13] with the addition of 125 μg of ganglioside mixture (Matreya, State College, PA, USA) in selected experiments as indicated. Briefly, GST and GST fusion proteins (150 pmol each) were immobilized to 10 μL of glutathione-sepharose-4B matrix (Qiagen, Hilden, Germany) and subsequently incubated for 2 h at 4 °C with 100 pmol H_C_ fragment in a total volume of 200 μL in binding buffer as stated in the respective figure legends. Beads were collected by centrifugation and washed two times each with the corresponding binding buffer. Washed pellet fractions were incubated at 37°C for 20 min in SDS sample buffer and analyzed by 12.5% SDS-PAGE. Protein bands were detected by Coomassie blue staining and subsequently quantified by densitometry using the software TINA (version 2.09f, Raytest, Straubenhardt, Germany). Unspecific binding of ligand to immobilized GST matrix was subtracted from the specific binding signal of H_C_.

### Assembly of phospholipid-bilayer nanodiscs

Nanodisc assembly was performed as described before with the following modifications [67]. Recombinant GST-rSyt-II 1–90 in PBS containing 0.5% Triton X-100 was spin-concentrated at 4000 × *g* for 20 min through Amicon Ultra-4 centrifugal filter units (Merck Millipore) to a concentration of approximately 3 to 4 mg/mL. A 100 mM POPC stock solution was prepared by drying lipids dissolved in chloroform in 6 mL borosilicate glass tubes (Fisher Scientific) under a stream of nitrogen and subsequently under vacuum for 4 hours before solving the dried lipids in a 400 mM sodium cholate solution in PBS by vortexing rigorously and ultrasonic treatment until a clear solution was obtained. Assembly mixtures of a total volume of 170 μL were prepared in glass tubes by adding 100 mM POPC, GT1b (10 μg/mL in PBS), concentrated GST-rSyt-II 1–90, and PBS to 100 μL of MSP 1E3D1. Depending on the concentration of the MSP stock solution used and the kind of assembled nanodiscs, the volume of the reagents was adjusted to fulfill the following criteria: for empty nanodiscs, 130 POPC molecules were added per two molecules MSP (130 lipids per nanodisc); for GT1b or dual-receptor nanodiscs the lipid content was reduced to 120 POPC molecules, while 10 molecules of GT1b were added per nanodiscs. Either 50 μL or 30.3 μL of concentrated GST-rSyt-II 1–90 was added for assembly into Syt-II nanodiscs or dual-receptor nanodiscs, respectively. The total volume was brought up to 170 μL by addition of PBS (pH 7.3) so that the final cholate concentration was between 26 to 28 mM at lipid concentrations between 6.5 and 7.0 mM. After incubating the mixtures for 30 min at room temperature, the self-assembly process was initiated by transferring the mixtures to 170 μg of Bio-Beads that have been washed with PBS and degassed. After 2 hours of incubation on a shaker at 4°C the mixtures were transferred to a second batch of Bio-Beads to remove effectively the high concentrations of Triton X-100 contained within the concentrated GST-rSyt-II stock solutions and incubated over-night at 4°C on a vertical shaker at 150 rpm. The next day, the assembled nanodiscs were transferred to a new glass tube and further purified and characterized by size exclusion chromatography (SEC). To this aim, the nanodiscs were fractioned using an ÄKTA Explorer 100 and a Superdex 200 Increase GL column (both GE Healthcare) at a flow rate of 0.75 mL/min in PBS. Beginning after 0.3 column volumes, 0.5-mL fractions were collected and further analyzed by indirect ELISA for the presence of receptor molecules integrated in nanodiscs.

### Characterization of phospholipid-bilayer nanodiscs by indirect ELISA

To identify SEC fractions containing both nanodiscs and receptor molecules, fractions were analyzed by indirect ELISA. To this aim, fractions were diluted 1:100 in PBS and 50 μL of this dilution were coated over-night to Nunc MaxiSorp microtiter plates (Thermo Fisher Scientific). The next day, the plates were washed with 4 × 300 μL of washing buffer (PBS containing 0.1% Tween 20) before being blocked for 2 hours at room temperature by adding 200 μL/well of a 3% (w/v) BSA solution in PBS. After washing, 50 μL of mouse anti-His (1:10,000), rabbit anti-synaptotagmin 2 (1:2500), or mouse anti-GT1b (1:2500) antibodies diluted in PBS containing 0.1% BSA were added for 1 hour at room temperature before bound antibodies were detected by incubation with 50 μL/well of HRP-coupled goat anti-mouse or rabbit IgG antibodies (used at 1:5000 or 1:3000 dilutions, respectively) after a further washing step. Signal development was initiated by adding 100 μL per well of Seramun Slow Blau TMB-substrate (Diavita) for 10 min before development was stopped by adding 0.25 M H_2_SO_4_. Finally, signals were read at 450 nm referenced to 620 nm using an Infinite 200 ELISA reader (Tecan). Fractions of the expected size for assembled nanodiscs were pooled and either used directly for kinetics measurements by SPR (empty nanodiscs, GT1b nanodiscs) or further purified using GST pull down (GST-rSyt-II and dual-receptor nanodiscs). Purified nanodiscs were stored at 4°C until used.

### Purification of receptor-containing nanodiscs by GST pull down

To separate nanodiscs containing recombinant GST-rSyt-II from nanodiscs without receptor proteins, we made use of the GST-tag contained on the Syt-II proteins for batch purification by GST pull down. To this aim, 334 μL of Protino glutathione agarose were added to glass tubes and washed once with 5 mL of PBS. After centrifugation for 5 min at 500 × *g* the supernatant was discarded and 1.75 mL of pooled nanodisc-containing fractions were added per tube. After a 1-hour incubation at room temperature under constant shaking (600 rpm) the glutathione agarose was pelleted by centrifugation and washed once with 5 mL of PBS. Bound nanodiscs were eluted by incubation with elution buffer (10 mM reduced glutathione in 50 mM Tris-HCl, pH 8.0) for 10 min at room temperature under shaking. Fractions before the purification (input), supernatant after binding (SN), and eluted nanodiscs (eluate) were collected and analyzed for the presence of nanodiscs and receptor molecules by SDS-PAGE, Coomassie staining, western and dot blot, and electron microscopy as described below.

### SDS-PAGE, Coomassie staining, and western and dot blot analysis of nanodiscs

For SDS-PAGE, fractions were mixed with 3 × Laemmli loading buffer containing dithiothreitol, heated for 5 min at 95°C, and cooled on ice before 10 μL were loaded on 12% polyacrylamide gels and separated according to standard procedures [68]. Gels were either stained using colloidal Coomassie staining [69] or electrophoretically transferred to methanol-activated PVDF membranes for subsequent western blotting [70]. To this aim, membranes were blocked for 1 hour at room temperature with 2% (w/v) skimmed milk powder in ELISA washing buffer before addition of either mouse anti-His (1:10,000) or rabbit anti-synaptotagmin 2 (1:5,000) antibodies. Detection was done by HRP-labelled goat anti-mouse IgG or anti-rabbit IgG (both 1:10,000) antibodies for 1 h at room temperature. All antibodies were diluted in blocking buffer and the membranes were washed between incubation steps for 3 × 5 min with washing buffer. Detection was done with SuperSignal West Dura Extended Duration Substrate (Thermo Fisher Scientific) on a ChemiDoc imaging system (Bio-Rad). Dot blots were performed accordingly except that 10 μL fractions of GT1b (100 μg/mL) were dropped on a nitrocellulose membrane and air-dried before proceeding with blocking and incubation with an anti-GT1b antibody (1:2,500) and HRP-labeled goat anti-mouse IgG antibodies (1:10,000).

### Electron microscopy

Suspensions of nanodiscs were prepared for negative staining electron microscopy using glow-discharged grids (400-mesh copper grid covered with carbon re-inforced plastic film). Uranylacetate (0.5%) was used as negative stain. Examination of samples was performed with the Tecnai 12 BioTwin (FEI, Thermo Fisher Scientific) transmission electron microscope operated at 120 kV, and images were recorded with an Eagle CCD camera (4096 x 4096 pixels, 16 bit; FEI, Thermo Fisher Scientific).

### Surface plasmon resonance (SPR) measurements

All SPR measurements were performed on a Biacore X100 or a T200 apparatus (GE Healthcare) using sensor chips CM5 and HBS-EP+ (10 mM HEPES, pH 7.4, 150 mM NaCl, 3 mM EDTA, 0.05% Tween 20) of HBS-N (10 mM HEPES, pH 7.4, 150 mM NaCl; nanodisc measurements) as running buffers at 25°C unless otherwise noted.

### Kinetic measurements of H_C_ binding to isolated protein receptor

Binding kinetics and affinity of recombinant receptor-binding domains of BoNT/B, DC, and G to GST-rSyt-II 1–61 were determined on a Biacore X100 as described previously [60]. Briefly, recombinant GST or GST-tagged rSyt-II were captured on flow cells 1 or 2 to immobilization densities of 100 resonance units (RUs) or 220 RUs, respectively, using a GST Capture Kit modified sensor chip (GE Healthcare) before 1:3 dilution series of recombinant receptor-binding domains (~50 kDa) were injected for 120 s at a flow rate of 30 μL/min, ranging from 1200 nM to 14.8 nM with duplicate injections at the highest concentration. The binding dissociation was monitored for 300 s before the sensor surface was regenerated using 10 mM glycine pH 2.1 for 120 s at a flow rate of 10 μL/min. All measurements were performed in duplicate.

### Thermodynamic analysis of BoNT/B receptor binding

To determine the thermodynamics of the interaction between H_C_B and GST-rSyt-II 1–61, both GST and GST-rSyt-II were immobilized covalently to a sensor chip CM5 each by dilution in 10 mM acetate buffer pH 4.5 (GE Healthcare) to 350 RUs (GST-rSyt-II) on flow cell 1 and 104 RUs (GST) on flow cell 2, using standard amine coupling chemistry (Amin coupling Kit; GE Healthcare) on a Biacore X100. Two-fold dilution series of H_C_B ranging from 800 nM to 6.25 nM with duplicate injections of the highest concentration were injected for 60 s before binding dissociation was monitored for 120 or 300 s. Regeneration was done by a 30 s injection of 10 mM glycine pH 1.7 at a flow rate of 10 μL/min. All measurements were replicated four times at 10°C, 15°C, 25°C, and 35°C.

### Kinetic and thermodynamic measurements of binding to nanodisc-incorporated receptor molecules

Nanodiscs containing no (empty nanodiscs), GT1b only, Syt-II only, or both receptor molecules (dual-receptor nanodiscs) were immobilized to a series S sensor chip CM5 (GE Healthcare) via the His-tag incorporated on the MSP. To this aim, the sensor surface was modified using the His Capture Kit (GE Healthcare) according to manufacturer’s recommendations. Empty nanodiscs diluted 1:20 in HBS-N buffer were immobilized to the control flow cells 1 or 3 for 120 s at 10 μL/min, leading to immobilization levels of 281 ±38 RUs. GT1b-containing nanodiscs (1:20), Syt-II-containing or dual-receptor nanodiscs (both 1:5) were immobilized accordingly to flow cells 2 or 4, leading to immobilization levels of 311 ±19, 409 ±30, and 275 ±25 RUs, respectively. After each capture step, the sensor chip was allowed to stabilize for 60 s before H_C_ fragments were injected at 66.6 nM, 200 nM, and 600 nM in a kinetic titration series (single cycle kinetics [71]) at a flow rate of 30 μL/min for 120 s followed by a 600-s injection of running buffer to monitor binding dissociation. The sensor surface was regenerated by injection of 10 mM glycine pH 1.5 for 60 s at a flow rate of 30 μL/min. For thermodynamic measurements, binding of H_C_B and H_C_B ΔG1247-F1250 to dual-receptor nanodiscs as well as H_C_B wild-type to Syt-II-only nanodiscs was repeated at 11°C, 15°C, 25°C, and 37°C. All measurements were performed in duplicate.

### Data analysis and curve fitting

All binding curves were double referenced as described [72]. Additionally, directly before each measurement, binding of recombinant receptor-binding domains to either GST or empty nanodiscs on both the control and measurement flow cell was determined. Double-referenced binding curves from these measurements, which arose due to partially ineffective regeneration of the sensor surface especially during later injection cycles, were additionally subtracted to prevent artefacts. Unless otherwise stated, all binding curves were fit to 1:1 Langmuir interaction models using the BIAevaluation software (4.1.1). Thermodynamic binding parameters ΔG°, ΔH°, and—TΔS° were derived from the temperature dependence of the binding affinity *K*_D_ by using van’t Hoff plots (S5 Fig). To this aim, ln (*K*_D_) was plotted over 1/T (Kelvin) and fitted using linear regression to determine the slope and Y-intercept using Prism 5.04 (GraphPad) from which ΔH° (= slope × gas constant *R*) and ΔS° (= Y-intercept ×–gas constant *R*) were calculated. ΔG° at 25°C was calculated from ΔH°–TΔS°.

## Supporting information

S1 Fig **A** Alignment of BoNT/B subtypes 1–8, BoNT/DC, and BoNT/G amino acid sequences covering the region of the hydrophobic HC loop (marked by boxes based on superimposition of crystal structures). Multiple sequence alignments were performed using Geneious 10.0.5 (global alignment with free end gaps employing the Blosum62 cost matrix) and the NCBI-Protein IDs denominated and visualized using the ENDscript server 3.0.1. Triangles in blue (BoNT/B), magenta (BoNT/G), or orange (BoNT/DC) mark amino acids at the tip of the H_C_ loop deleted in the Δloop mutants. **B** Crystal structures of H_C_B, H_C_DC, and H_C_G. The structures of H_C_B (dark blue ribbon; 2NM1.pdb), H_C_DC (purple; 4IRS.pdb) and H_C_G (beige; 2VWR.pdb) were superimposed. Syt-II peptide bound to H_C_DC is displayed as red α-helix and the key residue W1262 of the conserved GBS in H_C_B as orange sticks. The H_C_ loop comprises E1245-E1252 in H_C_B (light blue ribbon), F1245-H1255 in H_C_DC (grey ribbon), and K1250-D1257 in H_C_G (green ribbon, residues 1253–55 invisible due to flexible loop). Residues at the tip of the H_C_ loop deleted in H_C_B ΔG1247-F1250 and H_C_DC ΔY1251-F1253 are highlighted in light blue and purple sticks, respectively. **C** SDS-PAGE analysis of full-length BoNT/B, DC, and G wild-type and respective ΔH_C_ loop mutants as well as the corresponding H_C_ fragments. **D** Thermal denaturation CD analysis of wild-type and ΔH_C_ loop H_C_ fragments. All spectra revealed proteins rich in β-sheets which is in accordance with their known crystal structures (S1 Fig). Determination of the thermal stability of H_C_B and H_C_G wild-type yielded T_m_ values of 44.0°C and 46°C, respectively, confirming previous measurements [12]. Remarkably, H_C_DC wild-type displayed a 12°C higher T_m_ value than H_C_B wild-type. The T_m_ values of H_C_B ΔG1247-F1250, H_C_B I1248L/V1249L/F1250R, H_C_DC ΔY1251-F1253, and H_C_G ΔY1252-W1256 ranged by 1.5–3.0°C higher than their respective wild-type proteins. These minor increases clearly demonstrated that the secondary structures of the H_C_ ΔH_C_ loop mutants were not significantly altered due to the loop deletions.
(TIF)

S2 Fig Representative SDS-PAGE of GST pull down of wild-type and ΔH_C_ loop-mutants of H_C_B, H_C_DC, and H_C_G by GST-synaptotagmin-II with or without gangliosides in Triton X-100 micelles. Binding of 100 pmol of the wild-type or ΔH_C_ loop H_C_ fragments to 150 pmol GST, GST-rSyt-II 1–61, GST-rSyt-II 1–90 in 20 mM Tris pH 8, 80 mM NaCl, 0.5% Triton X-100 in the presence of 125 μg of ganglioside mix embedded in Triton X-100 micelles immobilized to glutathione-sepharose 4B matrix and subsequent SDS-PAGE analysis.
(TIF)

S3 Fig Additional purification of GST-rSyt-II containing nanodiscs via GST-pull down. Batch purification via the GST-tag using glutathione-agarose was used to separate receptor-bearing nanodiscs from empty nanodiscs. **A**. Colloidal Coomassie-stained gels for pooled nanodiscs containing fractions after SEC (input), supernatant (SN) of GST-pull-down material, and eluate from glutathione-agarose beads containing purified nanodiscs. **B** Western blot results using an anti-His antibody to detect MSP or an anti-Syt-II antibody to detect Syt-II. **C** Dot blot using an anti-GT1b antibody to check for GT1b in different fraction of GST-pull-down purification. Both empty and GT1b-containing nanodiscs were also tested in addition to GT1b not embedded in nanodiscs (GT1b ctrl).
(TIF)

S4 Fig Correlation between binding affinity determined by the indicated receptor molecules incorporated in Triton X-100 micelles by pull-down assay (given as Mol% binding) and incorporated in nanodiscs by SPR (given as Affinity in M). Highly significant (p < 0.0001) and close (Spearman r = -0.91) correlation between binding affinities determined by SPR and the mol% binding determined in the pull-down assays was observed for the interactions of both wild-type (closed symbols) and ΔH_C_ loop-mutant (open symbols) H_C_B (triangles), H_C_DC (squares), and H_C_G (circles). The mutant H_C_B I1248L/V1249L/F1250R comprising a B4-like H_C_ loop is shown with black borders.
(TIF)

S5 Fig Van’t Hoff plots for deduction of thermodynamic binding parameters. The natural logarithm (ln) of the binding affinities *K*_D_ was plotted over 1 divided by the measurement temperature for binding of H_C_B to isolated Syt-II (**A**), Syt-II incorporated into nanodiscs (**B**), and H_C_B ΔG1247-F1250 and H_C_B binding to dual-receptor nanodisc (**C** and **D**, respectively). Numbers indicate values from repeated measurements.
(TIF)

S6 Fig Binding specificity of the antibodies used in this study. Either 5 μL of rat brain extract or 562.5 ng of the indicated proteins were loaded on 12% PAA gels which were subsequently stained by colloidal Coomassie (**A**) or transferred to PVDF-membranes and probed with a mouse anti-His (1:10,000; **B**) or a rabbit anti-synaptotagmin 2 (1:5,000; **C**) antibody. **D**. To analyze the specificity of the mouse anti-GT1b antibody, 10 μL of the indicated proteins (diluted to 50 μg/mL in PBS) or gangliosides (100 μg/mL in PBS) were dripped on a nitrocellulose membrane, air-dried, and incubated with a 1:2,500 dilution of the antibody. All antibodies were highly specific for their respective target without any cross-reactivity against the other proteins or gangliosides tested.
(TIF)

S1 Table Binding kinetics and affinity data used to deduct thermodynamic binding parameters. Different values for same conditions indicate repeated measurements.
(DOCX)
