# Supplementary figures and images for: A lipid-binding loop of botulinum neurotoxin serotypes B, DC and G is an essential feature to confer their exquisite potency
Source: PLoS Pathog. 2018 May 2;14(5):e1007048. doi: 10.1371/journal.ppat.1007048 (PMC5951583; doi:10.1371/journal.ppat.1007048)

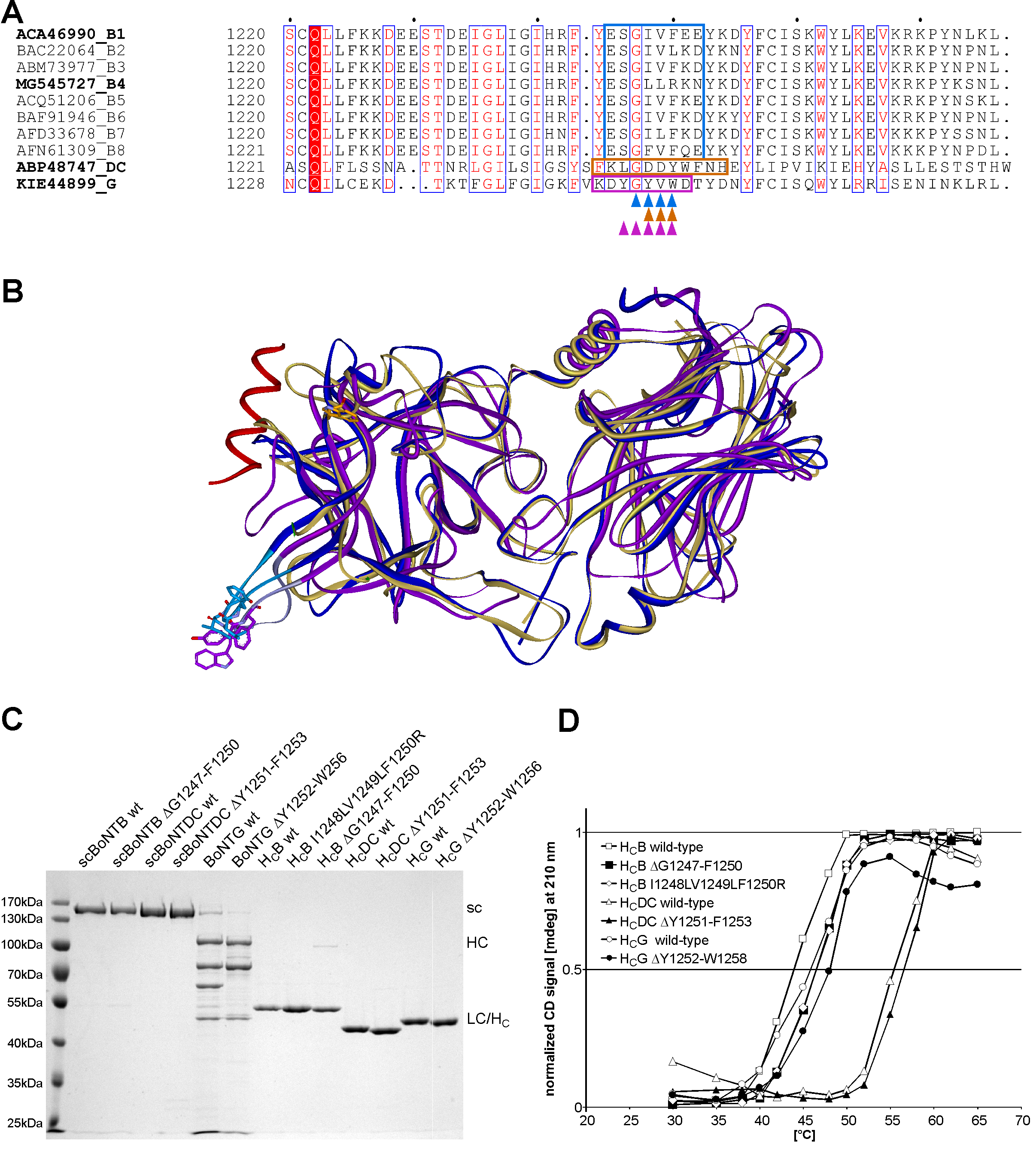

Supplement: S1 Fig — A Alignment of BoNT/B subtypes 1–8, BoNT/DC, and BoNT/G amino acid sequences covering the region of the hydrophobic HC loop (marked by boxes based on superimposition of crystal structures). Multiple sequence alignments were performed using Geneious 10.0.5 (global alignment with free end gaps employing the Blosum62 cost matrix) and the NCBI-Protein IDs denominated and visualized using the ENDscript server 3.0.1. Triangles in blue (BoNT/B), magenta (BoNT/G), or orange (BoNT/DC) mark amino acids at the tip of the HC loop deleted in the Δloop mutants. B Crystal structures of HCB, HCDC, and HCG. The structures of HCB (dark blue ribbon; 2NM1.pdb), HCDC (purple; 4IRS.pdb) and HCG (beige; 2VWR.pdb) were superimposed. Syt-II peptide bound to HCDC is displayed as red α-helix and the key residue W1262 of the conserved GBS in HCB as orange sticks. The HC loop comprises E1245-E1252 in HCB (light blue ribbon), F1245-H1255 in HCDC (grey ribbon), and K1250-D1257 in HCG (green ribbon, residues 1253–55 invisible due to flexible loop). Residues at the tip of the HC loop deleted in HCB ΔG1247-F1250 and HCDC ΔY1251-F1253 are highlighted in light blue and purple sticks, respectively. C SDS-PAGE analysis of full-length BoNT/B, DC, and G wild-type and respective ΔHC loop mutants as well as the corresponding HC fragments. D Thermal denaturation CD analysis of wild-type and ΔHC loop HC fragments. All spectra revealed proteins rich in β-sheets which is in accordance with their known crystal structures (S1 Fig). Determination of the thermal stability of HCB and HCG wild-type yielded Tm values of 44.0°C and 46°C, respectively, confirming previous measurements [12]. Remarkably, HCDC wild-type displayed a 12°C higher Tm value than HCB wild-type. The Tm values of HCB ΔG1247-F1250, HCB I1248L/V1249L/F1250R, HCDC ΔY1251-F1253, and HCG ΔY1252-W1256 ranged by 1.5–3.0°C higher than their respective wild-type proteins. These minor increases clearly demonstrated that the secondary structures of the HC ΔH [file ppat.1007048.s001.tif]

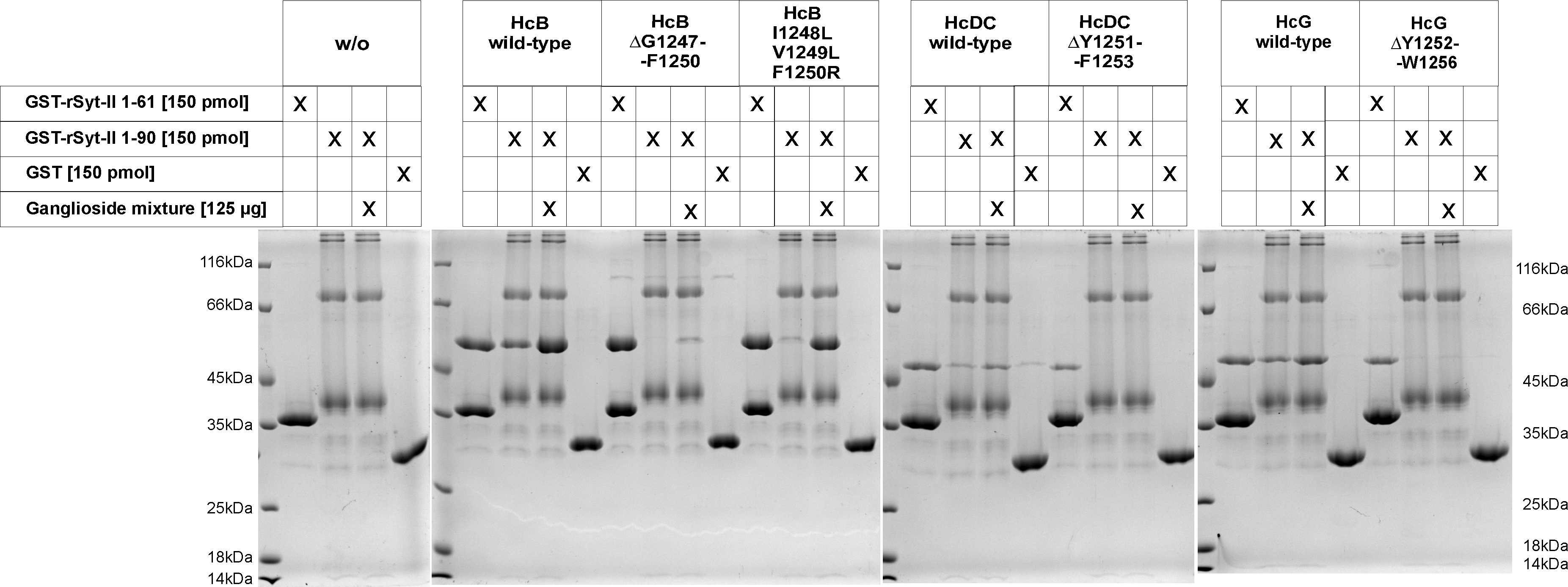

Supplement: S2 Fig — Binding of 100 pmol of the wild-type or ΔHC loop HC fragments to 150 pmol GST, GST-rSyt-II 1–61, GST-rSyt-II 1–90 in 20 mM Tris pH 8, 80 mM NaCl, 0.5% Triton X-100 in the presence of 125 μg of ganglioside mix embedded in Triton X-100 micelles immobilized to glutathione-sepharose 4B matrix and subsequent SDS-PAGE analysis. (TIF) [file ppat.1007048.s002.tif]

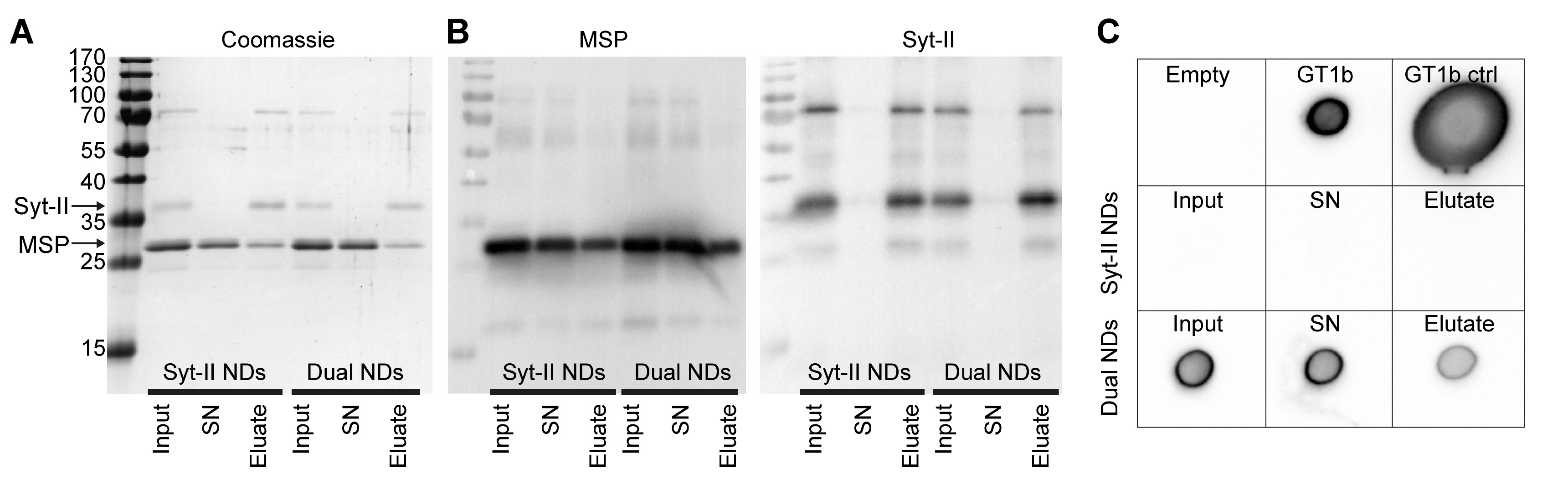

Supplement: S3 Fig — Batch purification via the GST-tag using glutathione-agarose was used to separate receptor-bearing nanodiscs from empty nanodiscs. A. Colloidal Coomassie-stained gels for pooled nanodiscs containing fractions after SEC (input), supernatant (SN) of GST-pull-down material, and eluate from glutathione-agarose beads containing purified nanodiscs. B Western blot results using an anti-His antibody to detect MSP or an anti-Syt-II antibody to detect Syt-II. C Dot blot using an anti-GT1b antibody to check for GT1b in different fraction of GST-pull-down purification. Both empty and GT1b-containing nanodiscs were also tested in addition to GT1b not embedded in nanodiscs (GT1b ctrl). (TIF) [file ppat.1007048.s003.tif]

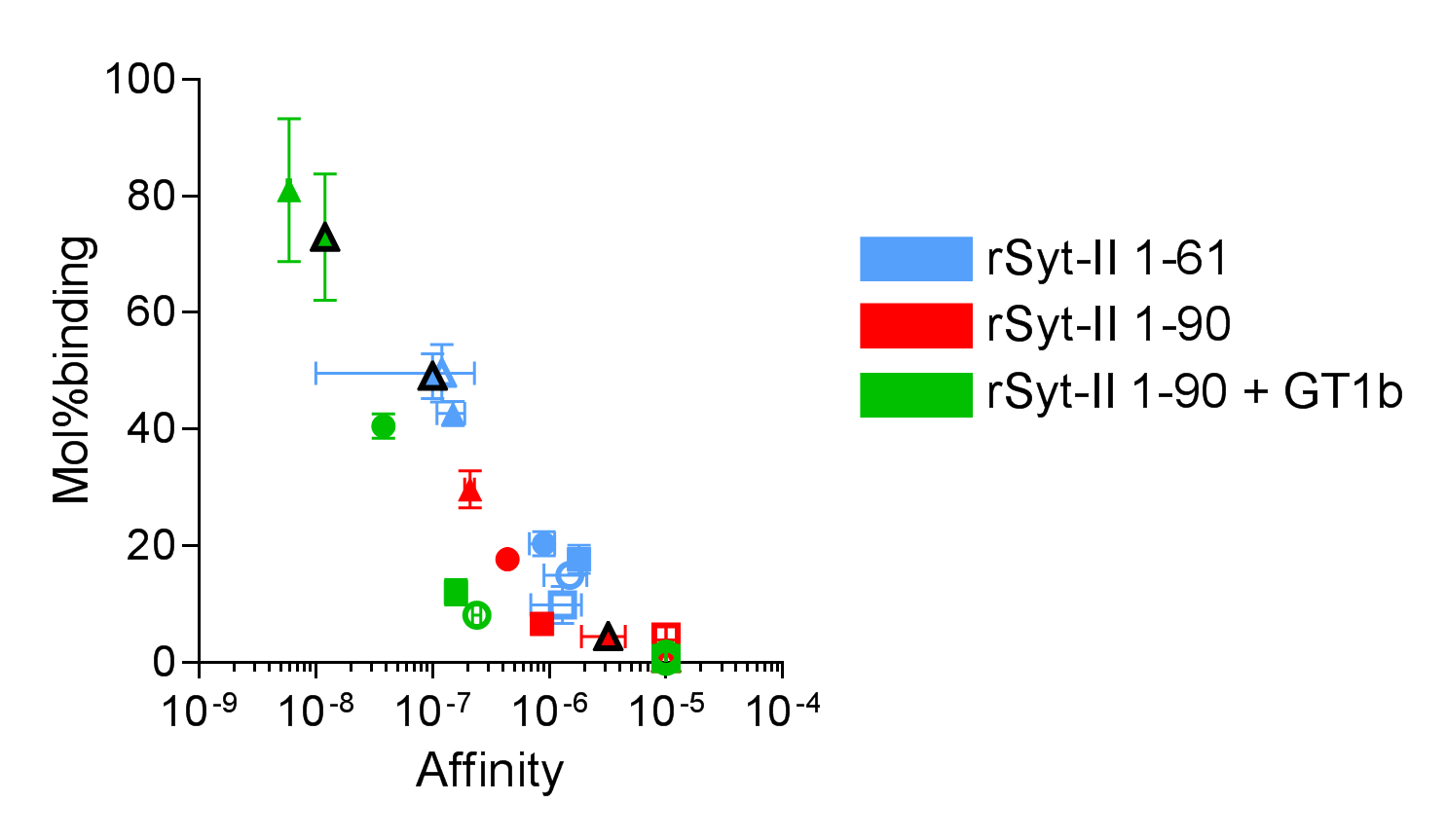

Supplement: S4 Fig — Highly significant (p < 0.0001) and close (Spearman r = -0.91) correlation between binding affinities determined by SPR and the mol% binding determined in the pull-down assays was observed for the interactions of both wild-type (closed symbols) and ΔHC loop-mutant (open symbols) HCB (triangles), HCDC (squares), and HCG (circles). The mutant HCB I1248L/V1249L/F1250R comprising a B4-like HC loop is shown with black borders. (TIF) [file ppat.1007048.s004.tif]

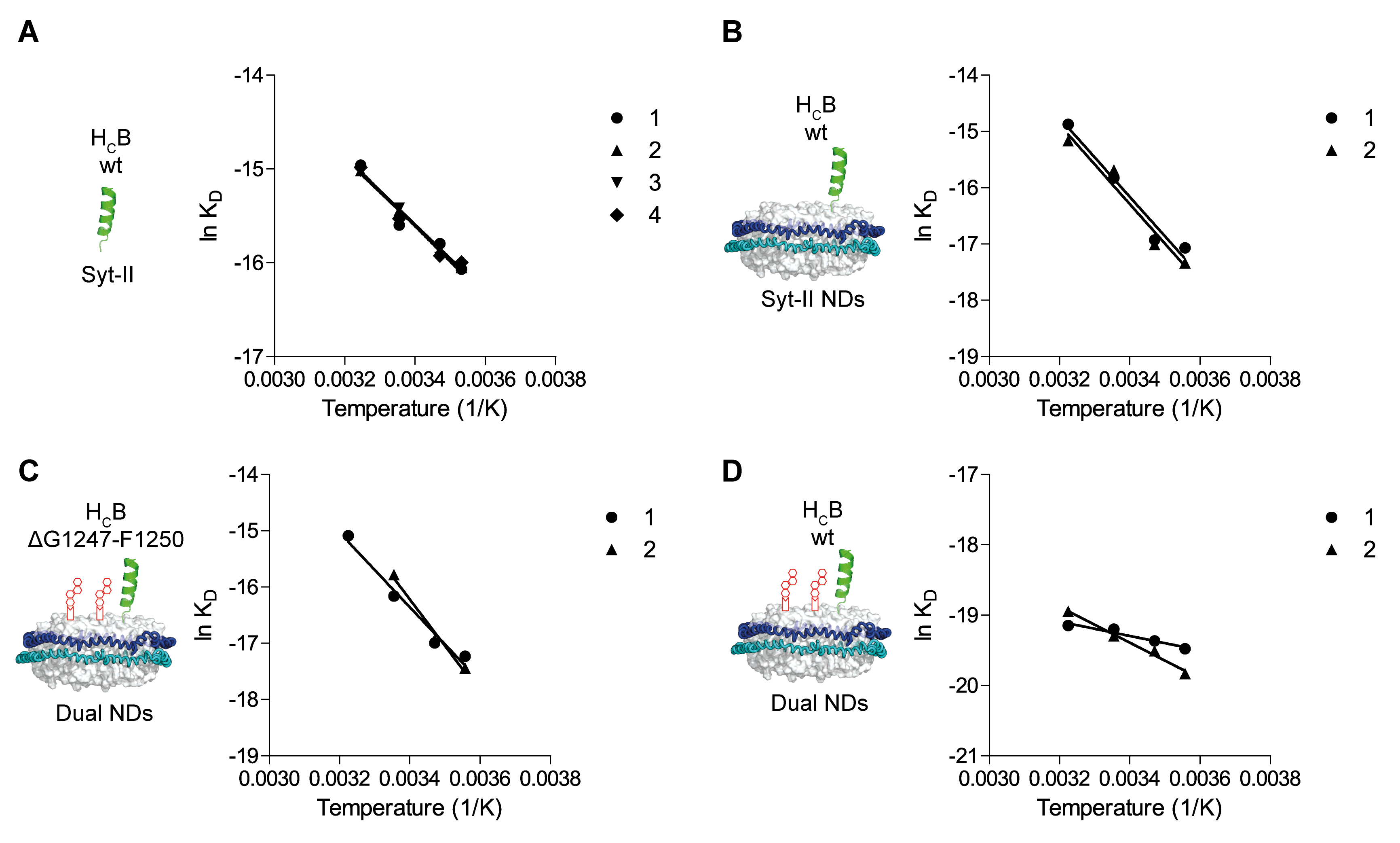

Supplement: S5 Fig — The natural logarithm (ln) of the binding affinities KD was plotted over 1 divided by the measurement temperature for binding of HCB to isolated Syt-II (A), Syt-II incorporated into nanodiscs (B), and HCB ΔG1247-F1250 and HCB binding to dual-receptor nanodisc (C and D, respectively). Numbers indicate values from repeated measurements. (TIF) [file ppat.1007048.s005.tif]

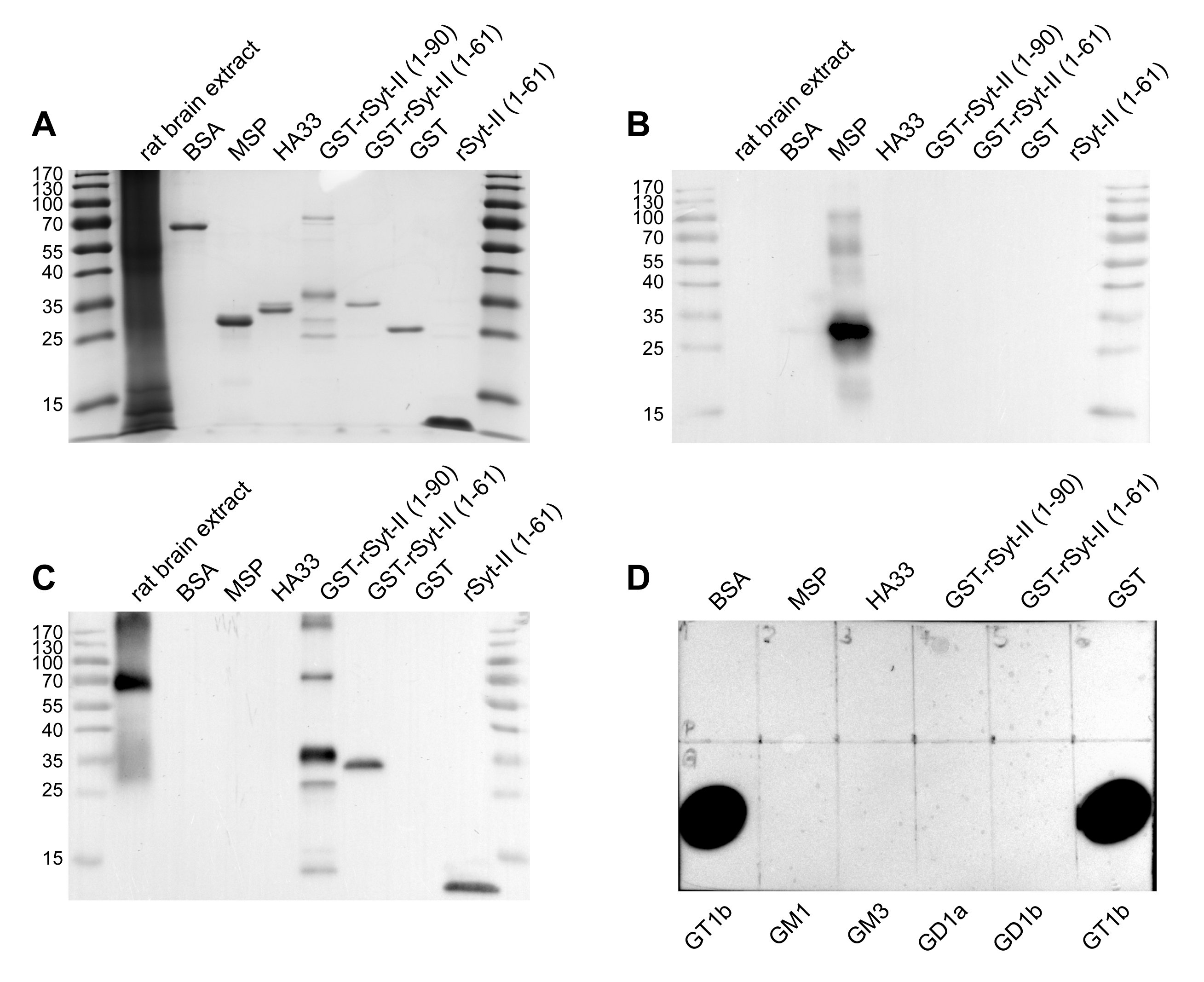

Supplement: S6 Fig — Either 5 μL of rat brain extract or 562.5 ng of the indicated proteins were loaded on 12% PAA gels which were subsequently stained by colloidal Coomassie (A) or transferred to PVDF-membranes and probed with a mouse anti-His (1:10,000; B) or a rabbit anti-synaptotagmin 2 (1:5,000; C) antibody. D. To analyze the specificity of the mouse anti-GT1b antibody, 10 μL of the indicated proteins (diluted to 50 μg/mL in PBS) or gangliosides (100 μg/mL in PBS) were dripped on a nitrocellulose membrane, air-dried, and incubated with a 1:2,500 dilution of the antibody. All antibodies were highly specific for their respective target without any cross-reactivity against the other proteins or gangliosides tested. (TIF) [file ppat.1007048.s006.tif]
