# Supplementary material for: A lipid-binding loop of botulinum neurotoxin serotypes B, DC and G is an essential feature to confer their exquisite potency
Source: PLoS Pathog. 2018 May 2;14(5):e1007048. doi: 10.1371/journal.ppat.1007048 (PMC5951583; doi:10.1371/journal.ppat.1007048)
Supplement: S1 Table — Different values for same conditions indicate repeated measurements. (DOCX) [file ppat.1007048.s007.docx]

**S1 Table. Binding kinetics and affinity data used to deduct thermodynamic binding parameters.** Different values for same conditions indicate repeated measurements.

| **Interaction (Receptor - Ligand)** | **Temperature (°C)** | **ka (1/Ms)** | **kd (1/s)** | **KD (M)** |
| --- | --- | --- | --- | --- |
| **GST-rSyt-II - H_C_B** | 10 | 2.4E+05 | 2.5E-02 | 1.1E-07 |
|  |  | 2.4E+05 | 2.5E-02 | 1.1E-07 |
|  |  | 2.7E+05 | 2.9E-02 | 1.1E-07 |
|  |  | 1.9E+05 | 2.2E-02 | 1.1E-07 |
|  | 15 | 2.9E+05 | 3.9E-02 | 1.4E-07 |
|  |  | 3.5E+05 | 4.4E-02 | 1.3E-07 |
|  |  | 3.3E+05 | 3.9E-02 | 1.2E-07 |
|  |  | 2.9E+05 | 3.6E-02 | 1.2E-07 |
|  | 25 | 6.5E+05 | 1.1E-01 | 1.7E-07 |
|  |  | 5.0E+05 | 9.9E-02 | 2.0E-07 |
|  |  | 4.8E+05 | 9.7E-02 | 2.0E-07 |
|  |  | 5.1E+05 | 9.2E-02 | 1.8E-07 |
|  | 35 | 6.3E+05 | 2.0E-01 | 3.2E-07 |
|  |  | 5.8E+05 | 1.7E-01 | 3.0E-07 |
|  |  | 5.9E+05 | 1.8E-01 | 3.0E-07 |
|  |  | 6.2E+05 | 1.9E-01 | 3.1E-07 |
| **Syt-II Nanodiscs - H_C_B** | 11 | 2.3E+04 | 9.0E-04 | 3.9E-08 |
|  |  | 3.7E+04 | 1.1E-03 | 3.0E-08 |
|  | 15 | 3.8E+04 | 1.7E-03 | 4.5E-08 |
|  |  | 5.1E+04 | 2.1E-03 | 4.1E-08 |
|  | 25 | 6.6E+04 | 8.8E-03 | 1.3E-07 |
|  |  | 5.6E+04 | 8.6E-03 | 1.5E-07 |
|  | 37 | 1.4E+05 | 4.8E-02 | 3.5E-07 |
|  |  | 2.1E+05 | 5.4E-02 | 2.6E-07 |
| **Dual Nanodiscs - H_C_B ΔH_C_ loop** | 11 | 7.3E+04 | 2.4E-03 | 3.3E-08 |
|  |  | 9.6E+04 | 2.6E-03 | 2.7E-08 |
|  | 15 | 1.3E+05 | 5.5E-03 | 4.2E-08 |
|  |  | 3.2E+05 | 1.4E-02 | 4.4E-08 |
|  | 25 | 8.8E+04 | 8.4E-03 | 9.6E-08 |
|  |  | 1.6E+05 | 2.3E-02 | 1.4E-07 |
|  | 37 | 1.5E+05 | 4.2E-02 | 2.8E-07 |
| **Dual Nanodiscs - H_C_B** | 11 | 7.0E+04 | 2.4E-04 | 3.5E-09 |
|  |  | 1.0E+05 | 2.5E-04 | 2.4E-09 |
|  | 15 | 8.4E+04 | 3.3E-04 | 3.9E-09 |
|  |  | 1.1E+05 | 3.8E-04 | 3.4E-09 |
|  | 25 | 1.4E+05 | 6.2E-04 | 4.6E-09 |
|  |  | 2.0E+05 | 8.3E-04 | 4.2E-09 |
|  | 37 | 1.1E+06 | 5.3E-03 | 4.8E-09 |
|  |  | 1.1E+07 | 6.5E-02 | 5.9E-09 |
